# Novel Method for Perceiving Key Requirements of Customer Collaboration Low-Carbon Product Design

**DOI:** 10.3390/ijerph15071446

**Published:** 2018-07-09

**Authors:** Aijun Liu, Qiuyun Zhu, Xiaohui Ji, Hui Lu, Sang-Bing Tsai

**Affiliations:** 1 Department of Management Engineering, School of Economics & Management, Xidian University, Xi’an 710071, China; xhji@stu.xidian.edu.cn; 2 State Key Laboratory for Manufacturing Systems Engineering, Xi’an Jiaotong University, Xi’an 710049, China; 3 Tianhua College, Shanghai Normal University, Shanghai 201815, China; janetluck@126.com; 4 Zhongshan Institute, University of Electronic Science and Technology, Zhongshan 528400, China

**Keywords:** low-carbon product design, customer collaborative product innovation, fuzzy grey relational analysis, genetic algorithm, green operation, green service, sustainability

## Abstract

Low-carbon product design is an important way to reduce greenhouse gas emission. Customer collaborative product innovation (CCPI) has become a new worldwide product design trend. Based on this popularity, we introduced CCPI into the low-carbon product design process. An essential step for implementing low carbon CCPI is to clarify key low carbon requirements of customers. This study tested a novel method for perceiving key requirements of customer collaboration low-carbon product design based on fuzzy grey relational analysis and genetic algorithm. Firstly, the study considered consumer heterogeneity, allowing different types of customers to evaluate low carbon requirements in appropriate formats that reflected their degrees of uncertainty. Then, a nonlinear optimization model was proposed to establish the information aggregation factor of customers based on the genetic algorithm. The weight of customers was obtained simultaneously. Next, the key low carbon requirements of customer were identified. Finally, the effectiveness of the proposed method was illustrated with a case related to a low carbon liquid crystal display.

## 1. Introduction

**Figure 1:**
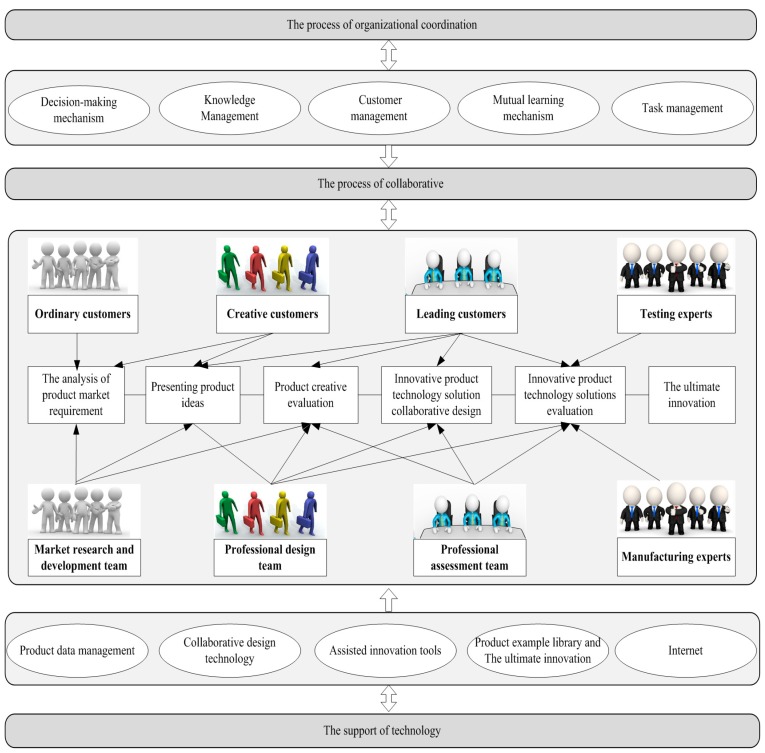
The Customer collaborative product innovation (CCPI) process.

With the continuous growth of the global population and economic scale, environmental problems caused by the use of energy are constantly recognized by people. Scientists have confirmed that rising atmospheric carbon dioxide (CO_2_) concentrations will bring about global climate change [1,2,3,4,5]. Many developing and developed countries use low-carbon products to achieve the long-term goal of reducing carbon dioxide emissions. Developed countries have laws and regulations to reduce emissions, such as the UK’s legally binding target of reducing its emissions by 80% by 2050 through the improvement of low-carbon supply chain technology, and there are similar laws and regulations in developing countries, such as China, India, and South Africa [6,7,8]. Decarburization has been emphasized as a significant strategy to respond to the environmental challenges created by climate change [9,10,11,12,13]. Wide customer use of low-carbon products is an important way to reduce carbon emissions. The use of low carbon products is an important means to reduce carbon dioxide emissions and promote public health. Low carbon preference of customers is an important index for enterprises to design low carbon products. If low carbon products do not consider consumer preferences, the market demand rate will be reduced, which restricts the promotion of low carbon products. Therefore, perceiving key requirements of customer collaboration low-carbon product design is crucial to protect the environment, reduce carbon emissions and thereby promote public health. However, the difference of customer education background, preference and knowledge lead to the evaluation information is fuzzy and the determination of key low-carbon requirements is difficult. Fuzzy refers to the uncertainty of customer evaluation information. Therefore, we propose a novel method to identify key low-carbon requirements of customers and to integrate sustainable development design concepts into the process of low-carbon product design. This approach can effectively enhance the communication between enterprises and customers, and improve enterprises performance. Lilien et al. compared and analyzed the customer collaborative product innovation (CCPI) process of 3M (Minnesota, Mining and Manufacturing) in the United States and illustrated the clear benefits of customer innovation with sales data [14]. The CCPI process is shown in Figure 1 [15]. The core of this method is identifying customers’ key low carbon requirements.

Methods for determining key low carbon requirements of customers include system model methods [16,17,18], mathematical model methods [19,20,21], and optimization algorithm methods [22,23]. Several system methods, such as Kano model, Analytic Hierarchy Process (AHP), user-centric approach, etc., are used in the existing literature. The Kano model uses consumer preferences to divide product attributes into five types: must-be, one-dimensional, attractive, indifferent, and reverse [24,25,26]. Yang et al. proposed a customer requirements acquisition system to gather customer requirements and instructed customers to express their requirements [27]. Chen et al. proposed an ontology learning customer requirements representation system, which pre-processes customer statements using language processing tools [28]. Wang and Tseng proposed the concept of customer requirements bias and used probability analysis methods to analyze customer requirements [29,30]. Liu et al. proposed a system management approach to manage requirements in industrial design [31]. Violante et al. developed a user-centric approach to meet a specific company’s requirements and help organizations effectively identify selection tools [32]. Sheng et al. studied the product service system, constructing a quality house and determined the attribute weight of the product and service [33]. Carulli et al. proposed a method for capturing customer requirements based on virtual reality technology. This technology is commonly used in the early stages of product design to establish customer requirements and reduce overall cost [34]. To solve the problem of inaccurate customer requirement, Kwong and Bai introduced the fuzzy number method based on the traditional analytic hierarchy process and proposed the fuzzy AHP to determine the importance of customer requirements [35]. The system model is simple to operate; however, it is a subjective approach and therefore does not always reflect the essential differences between items; the results tend to be more abstract. The application effect of this qualitative method is not always convincing.

Mathematical model methods, such as game theory approach, Quality Function Deployment (QFD), etc., are used in the existing literature. Li et al. combined the minimum deviation method, the Balanced Scorecard, the analytic hierarchy process, the proportional method, and other methods [36]. They proposed a system operation method that can make better use of product competition and preference information. Due to the ambiguity and uncertainty of customer requirements, Wang and Tseng established a probability-based Bayesian classifier using existing customer selection data, and classified customer requirements based on the flexibility of customer demand. Finally, they used a case study to show this method had clear advantages in customer demand classification [37]. Aguwa et al. developed a new approach to measure customer satisfaction by considering quantitative factors, including quantitative data, design parameters, drawing output, and decision-making templates as means of measurement [38]. This method can reduce errors and shorten the engineering development time. Liu et al. used a language intuitionistic fuzzy number to describe the decision maker’s language information. Then, the comparative analysis method was used to show the validity of the proposed method [39]. Nahm et al. proposed two methods of assessing customer preference and customer satisfaction. Assessing consumer preferences provided a way to capture incomplete and uncertain information about the customer; assessing consumer satisfaction involved building a customer satisfaction model based on competitive benchmarking [40]. The effectiveness of the proposed method was demonstrated using a door design example. Wu et al. integrated gray relational theory into the QFD [41]. This method accounts for the uncertainty and advancement of customer requirements, and is used to analyze dynamic customer requirements. Raharjo et al. proposed a method to address customer requirements’ dynamic in QFD [42]. Lo et al. proposed a one-step QFD to simplify traditional process, allowing users to meet special requirements [43]. Liu et al. presented an approach to address the dependent attribute problem, leading to a functional form with design attributes as independent variables [44]. This approach demonstrated the potential to optimize the design specification.

Many studies have indicated that customer requirement can be processed as an optimization problem [45,46,47,48,49] and heuristic method is commonly used to solve these problems. For example, genetic algorithm [45,46,47] and ant colony optimization (ACO) algorithm [48] have been widely applied to obtain a set of optimal solutions. Sagrado et al. used an ACO algorithm to analyze requirements [48]. To reduce uncertainty and the fuzzy feelings of customers, Song and Chan proposed the configuration optimization of a product-extension service (PES) [49].

The literature above indicates that many scholars have paid close attention to customer requirements, but have assumed that all customers have the same preference and have the same level of understanding about a product attribute. Besides, the system method is more subjective and cannot reflect the essential difference between items. The result of system method is not convincing. Mathematical methods are abstract problems of reality, but many problems cannot be quantitatively calculated accurately. Heuristic algorithm is often used to solve complex mathematical models, but many heuristic algorithms have shortcomings. Simply using the methods in the literature cannot solve the problem of perceiving key requirements of customer collaboration low-carbon product design very well. Therefore, it is necessary to consider the heterogeneity of customers. In addition, few studies integrate optimization algorithms with customer requirements. Thus, this study proposed a novel method for perceiving customer low carbon requirements to identify the key customer requirements in the process of CCPI. First, based on grey relational analysis (GRA), we defined the customer evaluation sequence, and addressed the hybrid fuzzy information associated with customers using an overall perspective. Then, a nonlinear optimization model was proposed to establish the information aggregation factor of customers, based on genetic algorithm. The weight of customers was obtained simultaneously. Finally, the study identified key low carbon requirements of customers.

The aim of this paper is to propose a novel method by considering consumer heterogeneity and allowing different types of customers to evaluate low carbon requirements in appropriate formats that reflect their degrees of uncertainty. This method can help enterprises accurately identify customers’ low carbon demand and greatly enhance their market competitiveness.

The remainder of this paper is organized as follows. Section 2 is the description of language information and grey relational analyses. Section 3 obtainss the key requirements of customers based on fuzzy grey relational analysis (FGRA). In Section 4, an empirical example is provided to demonstrate the applicability of the proposed method. Discussion is given in Section 5. Finally, some conclusions are summarized in Section 6.

## 2. Preliminaries

In the decision-making process, most linguistic models provide decision makers with a word to convey their preferences, using single linguistic terms. Decision makers may hesitate in selecting different linguistic terms as they work to express their preferences. As such, this study introduced a new approach to improve the accuracy of linguistic information in decision making, by using fuzzy numbers. This provides a novel way to structure linguistic expressions based on interval numbers, a triangular fuzzy number, and a trapezoidal fuzzy number. Then, we used grey relational analysis to deal with the customer evaluation language.

### 2.1. Interval Number

Interval number representation semantics means that each evaluation language corresponds to a part of the [0,1] interval, which is the control scope of the evaluation language, e.g., important and unimportant can be regard as evaluation language. If the evaluation language of the same language item set (e.g., *S*_0_, *S*_1_, …, *S*_g_) corresponds to the same interval length, then the language item set is a balanced language item set [50].

**Figure 2:**
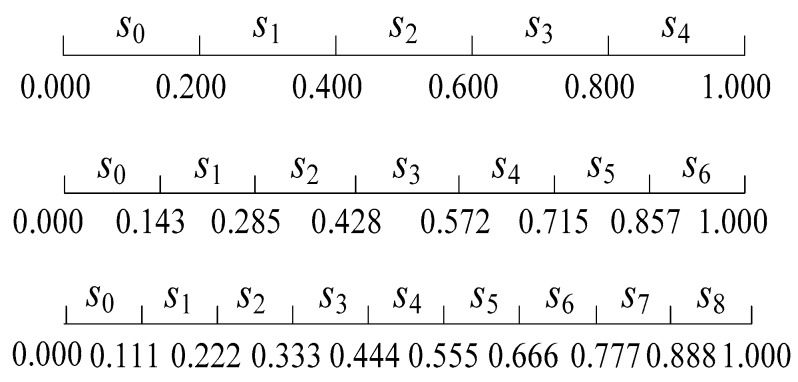
The interval number representation of five, seven and nine labels.

Figure 2 shows an example of a balanced language item set with five, seven and nine labels [51]. In addition, the relationship of language semantics and interval numbers is shown in Table 1.

**Definition** **1.** *The interval number is a set containing an interval of real numbers. It is represented by:*

$$A=[a^{L},a^{U}]$$

*where* $a^{L}$ *and* $a^{U}$ *are the lower and upper limits of the interval number, respectively.*

**Definition** **2.** *Let* $A=[a^{L},a^{U}]$ *and* $B=[b^{L},b^{U}]$ *be two interval numbers. Then, four operations are defined:*

*(1) Addition:*

(1) $$A+B=[a^{L},a^{U}]+[b^{L},b^{U}]=[a^{L}+b^{L},a^{U}+b^{U}]$$

*(2) Subtraction:*

(2) $$A-B=[a^{L},a^{U}]-[b^{L},b^{U}]=[a^{L}-b^{L},a^{U}-b^{U}]$$

*(3) Multiplication:*

(3) $$\begin{array}{ll} A\times B & =[a^{L},a^{U}]\times [b^{L},b^{U}]\\ & =[\min(a^{L}b^{L},a^{L}b^{U},a^{U}b^{L},a^{U}b^{U}),\max(a^{L}b^{L},a^{L}b^{U},a^{U}b^{L},a^{U}b^{U})] \end{array}$$

*(4) Division:*

(4) $$\begin{array}{ll} A/B & =[a^{L},a^{U}]/[b^{L},b^{U}]\\ & =\left(\min\left[\frac{a^{L}}{b^{U}},\frac{a^{L}}{b^{L}},\frac{a^{U}}{b^{U}},\frac{a^{U}}{b^{L}}\right],\max\left[\frac{a^{L}}{b^{U}},\frac{a^{L}}{b^{L}},\frac{a^{U}}{b^{U}},\frac{a^{U}}{b^{L}}\right]\right) \end{array}$$

### 2.2. Triangular Fuzzy Numbers

Some scholars defined triangular fuzzy numbers [52,53,54,55].

**Definition** **3** **[52].** *Let X be a reference set. The triangular fuzzy set* $\tilde{E}$ *of X is obtained through the expression* ${\tilde{h}}_{\bar{E}}(x)：\tilde{E}=\left\{\langle x,{\tilde{h}}_{\bar{E}}(x)\rangle |x\in X\right\}$*.* ${\tilde{h}}_{\bar{E}}(x)$ *is a set of triangular fuzzy numbers, and* $\tilde{h}={\tilde{h}}_{\bar{E}}(x)$ *represents the triangular fuzzy elements of* ${\tilde{h}}_{\bar{E}}(x)$*. If* $\tilde{\alpha }=\tilde{h}$*, then* $\tilde{\alpha }$ *is a triangular fuzzy numbers, which is donated by* $\tilde{\alpha }=\left({\tilde{\alpha }}^{L},{\tilde{\alpha }}^{M},{\tilde{\alpha }}^{U}\right)$*. That is* $\tilde{h}={\tilde{h}}_{\bar{E}}(x)=\left\{\left({\tilde{\alpha }}^{L},{\tilde{\alpha }}^{M},{\tilde{\alpha }}^{U}\right)|\tilde{\alpha }\in {\tilde{h}}_{\bar{E}}(x)\right\}$.

**Definition** **4** **[53].** *Let* $S=\left(s_{0},s_{1},\ldots ,s_{g}\right)$ *be a language term set;* $H_{s}=\left\{\langle x_{i},{\tilde{h}}_{s}(x_{i})\rangle |x_{i}\in X\right\}$ *be a fuzzy language set; and* $\tilde{h}={\tilde{h}}_{\bar{E}}(x)=\left\{\left({\tilde{\alpha }}^{L},{\tilde{\alpha }}^{M},{\tilde{\alpha }}^{U}\right)|\tilde{\alpha }\in {\tilde{h}}_{\bar{E}}(x)\right\}$ *be a triangular fuzzy set. The conversion function to transform a hesitant fuzzy set* $H_{s}$ *into a triangular fuzzy set* $\Delta (H_{s})$ *is defined as:*

(5) $$\Delta (H_{s})=\left\{\begin{array}{c} {\tilde{\alpha }}^{L}=\frac{\max(0,i-1)}{g}\\ {\tilde{\alpha }}^{M}=\frac{i}{g}\\ {\tilde{\alpha }}^{U}=\frac{\min(i+1,g)}{g} \end{array}\right\}$$

**Figure 3:**
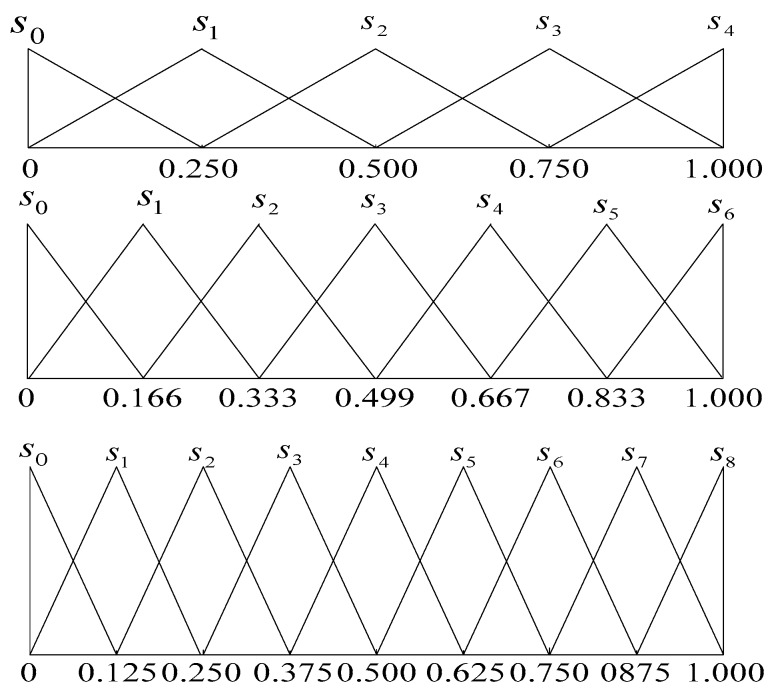
The triangular fuzzy number representation of five, seven and nine labels.

*where,* i=0,1,…,g*. According to these definitions, a set of language terms* $S=\left(s_{0},s_{1},\ldots ,s_{g}\right)$ *with labels g can be converted into the triangular fuzzy set* $\tilde{h}=\left\{\left(0,0,\frac{1}{g}),(0,\frac{1}{g},\frac{2}{g}),\ldots ,(\frac{i-2}{g},\frac{i-1}{g},\frac{i}{g}),\ldots ,(\frac{i-1}{g},1,1\right)\right\}$*. For realistic decision making, we commonly used five, seven and nine labels, and the process of transformation is shown in Figure 3. In addition, the relationship of language semantics and interval numbers is shown in Table 2.*

**Definition** **5** **[54].** *Let,* $\tilde{h}$*,* ${\tilde{h}}_{1}$ *and* ${\tilde{h}}_{2}$ *be three triangular fuzzy sets.* $\tilde{h}=\tilde{\alpha }=({\tilde{\alpha }}^{L},{\tilde{\alpha }}^{M},{\tilde{\alpha }}^{U})$ *,* ${\tilde{h}}_{1}={\tilde{\alpha }}_{1}=({\tilde{\alpha }}_{1}^{L},{\tilde{\alpha }}_{1}^{M},{\tilde{\alpha }}_{1}^{U})$*,* ${\tilde{h}}_{2}={\tilde{\alpha }}_{2}=({\tilde{\alpha }}_{2}^{L},{\tilde{\alpha }}_{2}^{M},{\tilde{\alpha }}_{2}^{U})$ *and* λ *is a constant. Then, the four operations are defined as:*

*(1) Addition:*

(6) $$\begin{array}{ll} {\tilde{h}}_{1}⊕{\tilde{h}}_{2} & =\left\{{\tilde{\alpha }}_{1}⊕{\tilde{\alpha }}_{2}|{\tilde{\alpha }}_{1}\in {\tilde{h}}_{1},{\tilde{\alpha }}_{2}\in {\tilde{h}}_{2}\right\}\\ & =\left\{({\tilde{\alpha }}_{1}^{L}+{\tilde{\alpha }}_{2}^{L}-{\tilde{\alpha }}_{1}^{L}{\tilde{\alpha }}_{2}^{L},{\tilde{\alpha }}_{1}^{M}+{\tilde{\alpha }}_{2}^{M}-{\tilde{\alpha }}_{1}^{M}{\tilde{\alpha }}_{2}^{M},{\tilde{\alpha }}_{1}^{U}+{\tilde{\alpha }}_{2}^{U}-{\tilde{\alpha }}_{1}^{U}{\tilde{\alpha }}_{2}^{U}|{\tilde{\alpha }}_{1}\in {\tilde{h}}_{1},{\tilde{\alpha }}_{2}\in {\tilde{h}}_{2}\right\} \end{array}$$

*(2) Subtraction:*

(7) $${\tilde{h}}_{1}⊗{\tilde{h}}_{2}=\left\{{\tilde{\alpha }}_{1}⊗{\tilde{\alpha }}_{2}|{\tilde{\alpha }}_{1}\in {\tilde{h}}_{1},{\tilde{\alpha }}_{2}\in {\tilde{h}}_{2}\right\}=\left\{({\tilde{\alpha }}_{1}^{L}{\tilde{\alpha }}_{2}^{L},{\tilde{\alpha }}_{1}^{M}{\tilde{\alpha }}_{2}^{M},{\tilde{\alpha }}_{1}^{U}{\tilde{\alpha }}_{2}^{U})|{\tilde{\alpha }}_{1}\in {\tilde{h}}_{1},{\tilde{\alpha }}_{2}\in {\tilde{h}}_{2}\right\}$$

*(3) Multiplication:*

(8) $$\lambda \tilde{h}=\left\{\lambda \tilde{\alpha }|\tilde{\alpha }\in \tilde{h}\right\}=\left\{(1-{\left(1-{\tilde{\alpha }}^{L}\right)}^{\lambda },(1-{\left(1-{\tilde{\alpha }}^{M}\right)}^{\lambda },(1-{\left(1-{\tilde{\alpha }}^{U}\right)}^{\lambda })|\tilde{\alpha }\in \tilde{h}\right\},\lambda >0$$

*(4) Division:*

(9) $${\tilde{h}}^{\lambda }=\left\{{\tilde{\alpha }}^{\lambda }|\tilde{\alpha }\in \tilde{h}\right\}=\left\{{\left({\tilde{\alpha }}^{L}\right)}^{\lambda },{\left({\tilde{\alpha }}^{M}\right)}^{\lambda },{\left({\tilde{\alpha }}^{U}\right)}^{\lambda })|\tilde{\alpha }\in \tilde{h}\right\},\lambda >0$$

**Definition** **6.** *Let* $\tilde{h}$*,* ${\tilde{h}}_{1}$ *and* ${\tilde{h}}_{2}$ *be three triangular fuzzy sets.* $\lambda ,\lambda_{1}$ *and* $\lambda_{2}$ *are constants greater than zero. This generates the following expressions:*

*(1) Addition:*

(10) $${\tilde{h}}_{1}⊕{\tilde{h}}_{2}={\tilde{h}}_{2}⊕{\tilde{h}}_{1};\;\lambda ({\tilde{h}}_{1}⊕{\tilde{h}}_{2})=\lambda {\tilde{h}}_{1}⊕\lambda {\tilde{h}}_{2}$$

*(2) Multiplication:*

(11) $$\left(\lambda_{1}\lambda_{2})\tilde{h}=\lambda_{1}(\lambda_{2}\tilde{h}\right);{\tilde{h}}_{1}⊗{\tilde{h}}_{2}={\tilde{h}}_{2}⊗{\tilde{h}}_{1};{\tilde{h}}_{1}{}^{\lambda }⊗{\tilde{h}}_{2}{}^{\lambda }={\left({\tilde{h}}_{2}⊗{\tilde{h}}_{1}\right)}^{\lambda };{\tilde{h}}^{\lambda_{1}\lambda_{2}}={\left({\tilde{h}}^{\lambda_{1}}\right)}^{\lambda_{2}}$$

**Definition** **7** **[55].** *Let* $\tilde{h}=({\tilde{\alpha }}^{L},{\tilde{\alpha }}^{M},{\tilde{\alpha }}^{U})$ *be a triangular fuzzy set, and* $s(\tilde{h})=\sum_{\tilde{\alpha }\in \tilde{h}}s(\tilde{\alpha })/\#\tilde{h}$ *be a scoring function of* $\tilde{h}$*, where* $\#\tilde{h}$ *is called the number of* $\tilde{h}$*.*

*Then,*

(12) $$s(\tilde{\alpha })=\frac{1}{3}({\tilde{\alpha }}^{L}+{\tilde{\alpha }}^{M}+{\tilde{\alpha }}^{U})$$

(13) $$sd(\tilde{\alpha })=\sqrt{\frac{1}{3}\left[{\left({\tilde{\alpha }}^{L}-s(\tilde{\alpha })\right)}^{2}+{\left({\tilde{\alpha }}^{M}-s(\tilde{\alpha })\right)}^{2}+{\left({\tilde{\alpha }}^{U}-s(\tilde{\alpha })\right)}^{2}\right]}$$

*For any two triangular fuzzy numbers* ${\tilde{h}}_{1}$ *and* ${\tilde{h}}_{2}$:

*(1)*  *If* $s({\tilde{h}}_{1})>s({\tilde{h}}_{2})$*, then* ${\tilde{h}}_{1}≻{\tilde{h}}_{2}$;
*(2)*  *If* $s({\tilde{h}}_{1})=s({\tilde{h}}_{2})$*, there are three cases:*
  *i.*  *if* $sd({\tilde{h}}_{1})>sd({\tilde{h}}_{2})$*, then* ${\tilde{h}}_{1}≺{\tilde{h}}_{2}$;
  *ii.*  *if* $sd({\tilde{h}}_{1})=sd({\tilde{h}}_{2})$*, then* ${\tilde{h}}_{1}={\tilde{h}}_{2}$; *and*
  *iii.*  *if* $sd({\tilde{h}}_{1})≺sd({\tilde{h}}_{2})$*, then* ${\tilde{h}}_{1}≻{\tilde{h}}_{2}$.

### 2.3. Trapezoidal Fuzzy Number

In many decision making circumstances, the use of linguistic information is in accurate. In these circumstances, we used trapezoidal fuzzy numbers to express the linguistic information. Other studies have defined trapezoidal fuzzy numbers [56,57,58].

**Definition** **8** **[59].** *Let* $S=\left(s_{0},s_{1},\ldots ,s_{g}\right)$ *be a language term set. The conversion function to translate the language term set S into a triangular fuzzy set* $\Delta (H_{s}^{T})$ *is defined as:*

(14) $$\Delta (H_{s}^{T})=(a_{i},b_{i},c_{i},d_{i})=\left\{\begin{array}{c} a_{i}=\max\left\{\frac{2i-1}{2g+1},0\right\}\\ b_{i}=\frac{2i}{2g+1}\\ c_{i}=\frac{2i+1}{2g+1}\\ d_{i}=\min\left\{\frac{2i+2}{2g+1},1\right\} \end{array}\right\}$$

**Figure 4:**
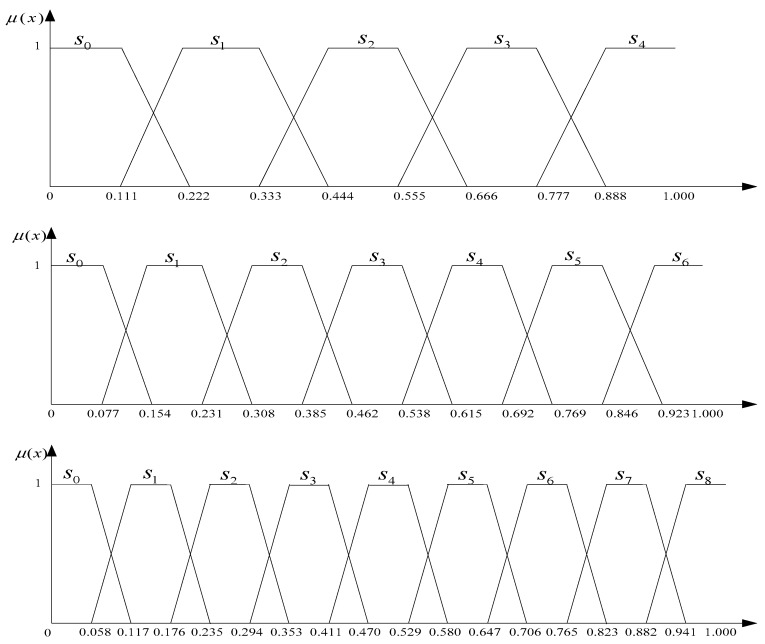
The trapezoidal fuzzy number representation of five, seven and nine labels.

*where* i=0,1,…,g*. When applying it to the common five, seven and nine labels, the process of transformation is shown in Figure 4.*

The semantics of the evaluation language is a quantitative description of evaluation language; it differs from the language itself. Therefore, we translate language semantics into trapezoidal intuitionist fuzzy numbers. The relationship of language semantics and trapezoidal fuzzy numbers is shown in Table 3.

Assume ${\tilde{A}}_{1}=(a_{1},b_{1},c_{1},d_{1})$ and ${\tilde{A}}_{2}=(a_{2},b_{2},c_{2},d_{2})$ are two trapezoidal fuzzy numbers and λ is a positive real number. Operations associated with the trapezoidal fuzzy numbers include the following:

(15) $${\tilde{A}}_{1}⊕{\tilde{A}}_{2}=(a_{1}+a_{2},b_{1}+b_{2},c_{1}+c_{2})$$

(16) $$\lambda {\tilde{A}}_{1}=(\lambda a_{1},\lambda b_{1},\lambda c_{1})$$

The variables $\tilde{A}=(a,b,c,d)$, ${\tilde{A}}_{1}=(a_{1},b_{1},c_{1},d_{1})$ and ${\tilde{A}}_{2}=(a_{2},b_{2},c_{2},d_{2})$ represent trapezoidal fuzzy numbers. The Murkowski distance $D_{p}({\tilde{A}}_{1},{\tilde{A}}_{2})$ between ${\tilde{A}}_{1}$ and ${\tilde{A}}_{2}$ is obtained using Equation (17):

(17) $$D_{p}({\tilde{A}}_{1},{\tilde{A}}_{2})={\left(\frac{{\left|a_{1}-a_{2}\right|}^{P}+2{\left|b_{1}-b_{2}\right|}^{P}+2{\left|c_{1}-c_{2}\right|}^{P}+{\left|d_{1}-d_{2}\right|}^{P}}{6}\right)}^{1/p}$$

There are three possible levels or definitions satiations for the value of *P*:

When p≥1, *P* represents the distance parameter.

When p=1, $D_{p}({\tilde{A}}_{1},{\tilde{A}}_{2})$ represents the Manhattan distance, which is denoted by the following formula:

(18) $$D_{p}({\tilde{A}}_{1},{\tilde{A}}_{2})=\frac{\left|a_{1}-a_{2}\right|+2\left|b_{1}-b_{2}\right|+2\left|c_{1}-c_{2}\right|+\left|d_{1}-d_{2}\right|}{6}$$

When p=2, $D_{p}({\tilde{A}}_{1},{\tilde{A}}_{2})$ stands for weighted Euclidean distance, that is

(19) $$D_{p}({\tilde{A}}_{1},{\tilde{A}}_{2})=\sqrt{\frac{{\left|a_{1}-a_{2}\right|}^{2}+2{\left|b_{1}-b_{2}\right|}^{2}+2{\left|c_{1}-c_{2}\right|}^{2}+{\left|d_{1}-d_{2}\right|}^{2}}{6}}$$

As a special case, when *b* = *c*, the trapezoidal fuzzy number is decomposed to triangular fuzzy numbers.

### 2.4. Grey Relational Analyses

**Figure 5:**
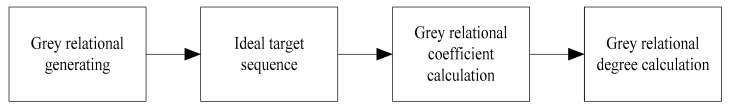
The grey relational analysis process.

Grey theory is an effective method for decision making. It was proposed by Deng to conduct systems analysis despite having incomplete information [60]. Gray relationships refer to the uncertain relationship between things, system elements, or between elements and behaviors. The grey relational analysis is a quantitative analysis, or evaluation of alternatives. It is widely used in many fields, including systems analysis, modeling and forecasting, and multiple attribute decision making problems, especially when handling unknown or incomplete information. The primary advantages of the GRA method as a decision-making method is that decisions are not complicated to calculate, and the consequences are based on original data. The main GRA process converts attribute values of all alternatives into comparable sequences, by removing the effects of different dimensions. Based on these sequences, we define a reference sequence. Then, we obtain the gray relationship coefficient. Finally, based on these grey correlation coefficients, we calculate the grey correlation level. Figure 5 shows the grey relational analysis process [61].

## 3. Method

**Figure 6:**
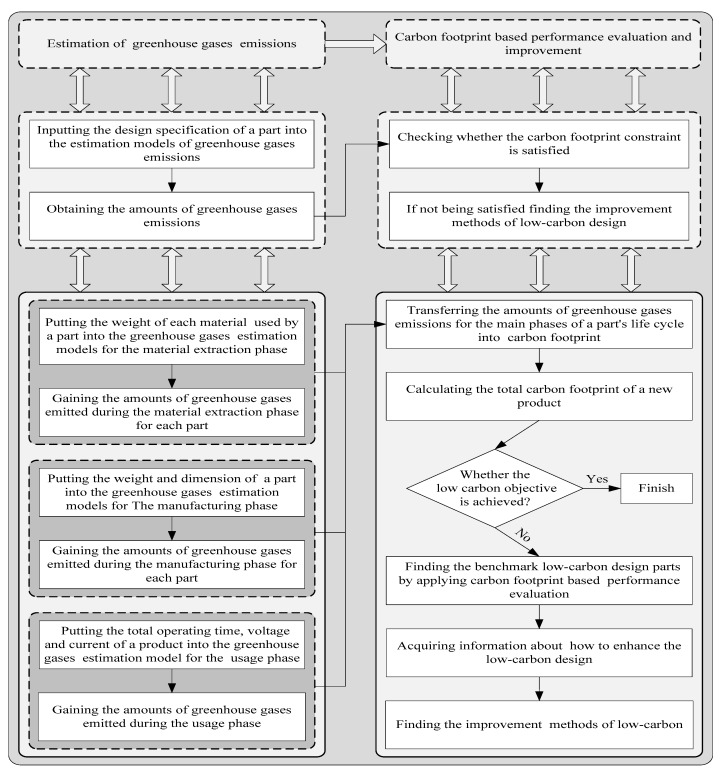
The process for low-carbon product design.

Due to resource restrictions, such as technology, cost, and equipment, designers cannot meet all low carbon requirements [62,63]. According to the process shown in Figure 6, we propose an optimization method combining a fuzzy grey relational analysis method with customer collaboration low-carbon product design to estimate low carbon economy efficiency. In this process, the core goal is determining the customers’ key low carbon requirements.

Acquiring key customer requirements is critical for the successful collaborative design of low-carbon products. Different consumers have different educational backgrounds, product experiences, professional knowledge, and judgment abilities. This leads to a significant degree of uncertainty in evaluating product information. Therefore, in the process of CCPI for low-carbon products, consumer weights cannot be considered equal. Based on consumer heterogeneity, we use a hybrid customer evaluation information representation method to perform a grey correlation analysis of customer evaluation information. This ultimately determines key customer requirements for low-carbon products.

A company wants to develop a new low carbon product using the customer collaborative product design method. To help product design engineers efficiently and effectively develop low-carbon products, the life cycle assessment method is used to analyze the carbon footprint of each phase. Figure 6 shows the specific process [62]. Finally, *M* customer requirements were determined that more significantly impact carbon emissions.

First, establish the language term set, and allow all customers to evaluate their requirements for low-carbon products. Different customers have different understanding of the performance of low-carbon products. Therefore, we use interval numbers, triangular fuzzy numbers and trapezoidal fuzzy numbers to describe the language evaluation information of leading customers, creative customers and ordinary customers, respectively. The specific form of expression is shown in Table 4.

**Figure 7:**
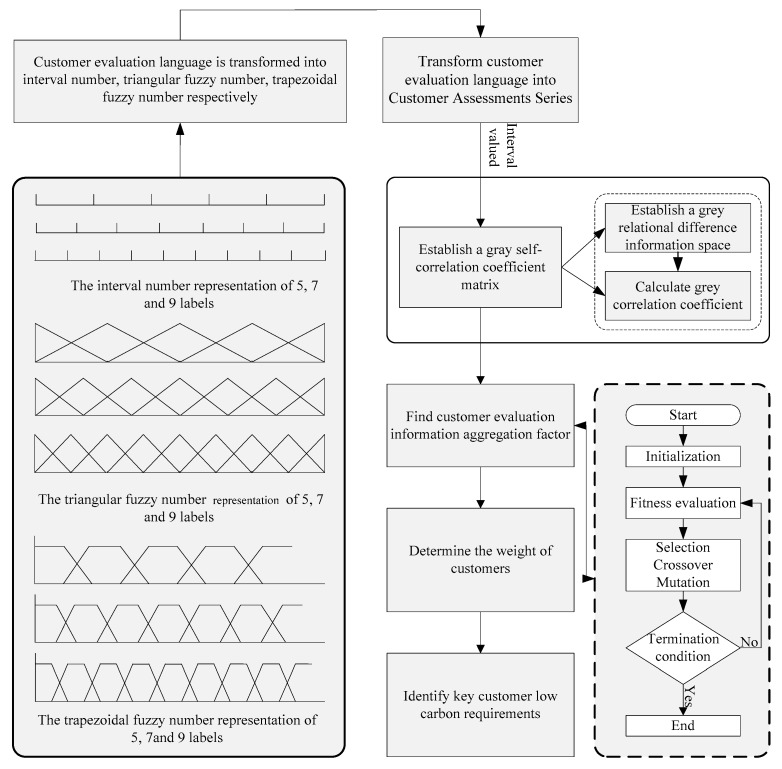
The proposed method’s process.

The steps of fuzzy grey relational analysis are shown in Figure 7.

**Step 1.** Transform the customer evaluation language into a Customer Assessment Sequence (CAS).

The assessment sequence $CAS_{k}$ consists of evaluation information of all customers for customer requirements. The $CAS_{k}$ of $C_{k}$ is donated by $CAS_{k}=\left(a_{k}\left(1\right),a_{k}\left(2\right),\ldots ,a_{k}\left(m\right),\ldots ,a_{k}\left(M\right)\right)$. The expression $a_{k}\left(m\right)$ can be an interval number, triangular fuzzy number, or trapezoidal fuzzy number.

Because there is a diverse degree of uncertainty as different customers evaluate low carbon product requirements, the form of evaluation information is also different. To compare the *CAS_k_*, different forms of evaluation information should be standardized. Therefore, we define a gray sequence generation operation to address interval numbers, triangular fuzzy numbers and trapezoidal fuzzy numbers. This achieves the transformation between different forms of *CAS_k_* and Normal Customer Assessment Sequences (NCASs).

Let $CAS_{k}=\left(a_{k}^{I}\left(1\right),a_{k}^{I}\left(2\right),\ldots ,a_{k}^{I}\left(m\right),\ldots ,a_{k}^{I}\left(M\right)\right)$ be the assessments series of $C_{k}$. $a_{k}^{I}\left(m\right)=\left(a_{k}^{L}\left(m\right),a_{k}^{U}\left(m\right)\right)$ is the interval number [64].

(20) $$CAS_{k}^{v}=CAS_{k}D_{1}=\left(a_{k}^{I}\left(1\right)d_{1},a_{k}^{I}\left(2\right)d_{1},\ldots ,a_{k}^{I}\left(m\right)d_{1},\ldots ,a_{k}^{I}\left(M\right)d_{1}\right)$$

In this expression,

(21) $$\begin{array}{ll} a_{k}^{v}(m) & =a_{k}^{I}\left(m\right)d_{1}=\frac{a_{k}^{I}\left(m\right)-\underset{m}{\min}a_{k}^{I}\left(m\right)}{\underset{m}{\max}a_{k}^{I}\left(m\right)-\underset{m}{\min}a_{k}^{I}\left(m\right)}\\ & =\sqrt{\frac{{\left(a_{k}^{L}\left(m\right)-\underset{m=1,2,\ldots ,M}{\min}\left\{a_{k}^{L}\left(m\right)\right\}\right)}^{2}+{\left(a_{k}^{U}\left(m\right)-\underset{m=1,2,\ldots ,M}{\min}\left\{a_{k}^{U}\left(m\right)\right\}\right)}^{2}}{{\left(\underset{m=1,2,\ldots ,M}{\max}\left\{a_{k}^{L}\left(m\right)\right\}-\underset{m=1,2,\ldots ,M}{\min}\left\{a_{k}^{L}\left(m\right)\right\}\right)}^{2}+{\left(\underset{m=1,2,\ldots ,M}{\max}\left\{a_{k}^{U}\left(m\right)\right\}-\underset{m=1,2,\ldots ,M}{\min}\left\{a_{k}^{U}\left(m\right)\right\}\right)}^{2}}} \end{array}$$

where m=1,2,…,M represents the number of customer requirements; *D*_1_ is the interval-valued operator of the interval number; and $a_{k}^{v}(m)$ represents the normal evaluation value of the importance of low-carbon *CR_m_* after interval valued processing.

Let $CAS_{k}=\left(a_{k}^{S}\left(1\right),a_{k}^{S}\left(2\right),\ldots ,a_{k}^{S}\left(m\right),\ldots ,a_{k}^{S}\left(M\right)\right)$ be the assessment sequence of $C_{k}$. $a_{k}^{S}\left(m\right)=\left(a_{k}^{L}\left(m\right),a_{k}^{M}\left(m\right),a_{k}^{U}\left(m\right)\right)$ is triangular fuzzy number [64].

(22) $$CAS_{k}^{v}=CAS_{k}D_{2}=\left(a_{k}^{S}\left(1\right)d_{2},a_{k}^{S}\left(2\right)d_{2},\ldots ,a_{k}^{S}\left(m\right)d_{2},\ldots ,a_{k}^{S}\left(M\right)d_{2}\right)$$

In this expression,

(23) $$a_{k}^{v}(m)=a_{k}^{S}\left(m\right)d_{2}=\frac{a_{k}^{S}\left(m\right)-\underset{m}{\min}a_{k}^{S}\left(m\right)}{\underset{m}{\max}a_{k}^{S}\left(m\right)-\underset{m}{\min}a_{k}^{S}\left(m\right)}\;=\sqrt{\frac{\begin{array}{l} {\left(a_{k}^{L}\left(m\right)-\underset{m=1,2,\ldots ,M}{\min}\left\{a_{k}^{L}\left(m\right)\right\}\right)}^{2}+{\left(a_{k}^{M}\left(m\right)-\underset{m=1,2,\ldots ,M}{\min}\left\{a_{k}^{M}\left(m\right)\right\}\right)}^{2}\\ +{\left(a_{k}^{U}\left(m\right)-\underset{m=1,2,\ldots ,M}{\min}\left\{a_{k}^{U}\left(m\right)\right\}\right)}^{2} \end{array}}{\begin{array}{l} {\left(\underset{m=1,2,\ldots ,M}{\max}\left\{a_{k}^{L}\left(m\right)\right\}-\underset{m=1,2,\ldots ,M}{\min}\left\{a_{k}^{L}\left(m\right)\right\}\right)}^{2}+{\left(\underset{m=1,2,\ldots ,M}{\max}\left\{a_{k}^{M}\left(m\right)\right\}-\underset{m=1,2,\ldots ,M}{\min}\left\{a_{k}^{M}\left(m\right)\right\}\right)}^{2}\\ +{\left(\underset{m=1,2,\ldots ,M}{\max}\left\{a_{k}^{U}\left(m\right)\right\}-\underset{m=1,2,\ldots ,M}{\min}\left\{a_{k}^{U}\left(m\right)\right\}\right)}^{2} \end{array}}}$$

where m=1,2,…,M represents the number of customer requirements; *D*_2_ is the interval-valued operator of the triangular fuzzy number; and $a_{k}^{v}(m)$ indicates the normal evaluation value importance of low-carbon *CR_m_* after interval valued processing.

Let $CAS_{k}=\left(a_{k}^{T}\left(1\right),a_{k}^{T}\left(2\right),\ldots ,a_{k}^{T}\left(m\right),\ldots ,a_{k}^{T}\left(M\right)\right)$ be the assessment sequence of $C_{k}$. $a_{k}^{T}\left(m\right)=\left(a_{k}^{L}\left(m\right),a_{k}^{M}\left(m\right),a_{k}^{N}\left(m\right),a_{k}^{U}\left(m\right)\right)$ is a trapezoidal fuzzy number [64].

(24) $$CAS_{k}^{v}=CAsS_{k}D_{3}=\left(a_{k}^{T}\left(1\right)d_{3},a_{k}^{T}\left(2\right)d_{3},\ldots ,a_{k}^{T}\left(m\right)d_{3},\ldots ,a_{k}^{T}\left(M\right)d_{3}\right)$$

In this expression,

(25) $$\begin{array}{ll} a_{k}^{v}(m) & =a_{k}^{T}\left(m\right)d_{3}=\frac{a_{k}^{T}\left(m\right)-\underset{m}{\min}a_{k}^{T}\left(m\right)}{\underset{m}{\max}a_{k}^{T}\left(m\right)-\underset{m}{\min}a_{k}^{T}\left(m\right)}\\ & =\sqrt{\frac{\begin{array}{l} {\left(a_{k}^{L}\left(m\right)-\underset{m=1,2,\ldots ,M}{\min}\left\{a_{k}^{L}\left(m\right)\right\}\right)}^{2}+{\left(a_{k}^{M}\left(m\right)-\underset{m=1,2,\ldots ,M}{\min}\left\{a_{k}^{M}\left(m\right)\right\}\right)}^{2}+\\ {\left(a_{k}^{N}\left(m\right)-\underset{m=1,2,\ldots ,M}{\min}\left\{a_{k}^{N}\left(m\right)\right\}\right)}^{2}+{\left(a_{k}^{U}\left(m\right)-\underset{m=1,2,\ldots ,M}{\min}\left\{a_{k}^{U}\left(m\right)\right\}\right)}^{2} \end{array}}{\begin{array}{l} {\left(\underset{m=1,2,\ldots ,M}{\max}\left\{a_{k}^{L}\left(m\right)\right\}-\underset{m=1,2,\ldots ,M}{\min}\left\{a_{k}^{L}\left(m\right)\right\}\right)}^{2}+{\left(\underset{m=1,2,\ldots ,M}{\max}\left\{a_{k}^{M}\left(m\right)\right\}-\underset{m=1,2,\ldots ,M}{\min}\left\{a_{k}^{M}\left(m\right)\right\}\right)}^{2}+\\ {\left(\underset{m=1,2,\ldots ,M}{\max}\left\{a_{k}^{N}\left(m\right)\right\}-\underset{m=1,2,\ldots ,M}{\min}\left\{a_{k}^{N}\left(m\right)\right\}\right)}^{2}+{\left(\underset{m=1,2,\ldots ,M}{\max}\left\{a_{k}^{U}\left(m\right)\right\}-\underset{m=1,2,\ldots ,M}{\min}\left\{a_{k}^{U}\left(m\right)\right\}\right)}^{2} \end{array}}} \end{array}$$

where *D*_3_ is the interval-valued operator of the trapezoidal fuzzy number; and $a_{k}^{v}(m)$ indicates the normal evaluation value importance of low-carbon *CR_m_* after interval valued processing.

**Step 2.** Build a gray self-correlation coefficient matrix.

In the process of GRA, when the reference sequence is known, considering the diversity of CAS for different customers, we construct a gray self-correlation coefficient matrix to describe the differences between different CAS.

(1) Establish a grey relational difference information space

Let $\Delta_{kj}(m)$ be the different information between $C_{k}$ and $C_{j}$ to evaluate $CR_{m}$. Then, there is different information set Δ:

(26) $$\Delta =\left\{\Delta_{kj}(m)=\left|a_{k}^{v}(m)-a_{j}^{v}(m)\right|,a_{k}^{v}(m)=a_{k}(m)d_{l},a_{k}^{v}(m)=a_{j}(m)d_{l}\right\}$$

(27) $$\Delta_{GR}=(\Delta ,\rho ,E_{1}^{\max},E_{1}^{\min})$$

where k,j=1,2,…Kand k≠j;m=1,2,…M, l=1,2,3 $\Delta_{kj}=\left(\Delta_{kj}(1),\Delta_{kj}(2),\ldots ,\Delta_{kj}(m)\right)$ $E_{1}^{\max}=\underset{k}{\max}\;\underset{j}{\max}\;\underset{m}{\max}\;\Delta_{kj}(m)$ represents the upper environmental parameters; $E_{1}^{\min}=\underset{k}{\min}\;\underset{j}{\min}\;\underset{m}{\min}\;\Delta_{kj}(m)$ represents the lower environmental parameters; *ρ* is resolution coefficient; and $\Delta_{GR}$ represents the grey relational difference information space.

(2) Calculate the grey correlation coefficient

The grey correlation coefficient is mainly used to measure the closeness of $C_{k}$ and $C_{j}$ when evaluating $CR_{m}$. When the grey correlation coefficient is larger, the $a_{k}^{v}(m)$ and $a_{j}^{v}(m)$ are closer. The difference in the evaluation of $C_{k}$ and $C_{j}$ indicates that the $CR_{m}$ is small. We can obtain the gray correlation coefficient using Equation (28):

(28) $$\theta \left(a_{k}^{v}(m),a_{j}^{v}(m)\right)=\theta \left(a_{j}^{v}(m),a_{k}^{v}(m)\right)=\frac{E_{1}^{\min}+\rho E_{1}^{\max}}{\Delta_{kj}(m)+\rho E_{1}^{\max}},k,j=1,2,\ldots K\;and\;k\neq j$$

In Equation (28), $\theta \left(a_{k}^{v}(m),a_{j}^{v}(m)\right)$ is the gray correlation coefficient between $a_{k}^{v}(m)$ and $a_{j}^{v}(m)$; and ρ∈[0,1] is a distinguishing coefficient, with a general value of 0.5.

(3) Build a gray self-correlation coefficient matrix

This step involves building a grey self-correlation coefficient matrix $R={\left[\left(\theta \left(a_{k}^{v}(m),a_{j}^{v}(m)\right)\right)\right]}_{\frac{K(K-1)}{2}\times M}$ by calculating the grey correlation coefficient of customer $C_{k}$ and customer $C_{j}$ with respect to customer requirement $CR_{m}$.

(29) $$R={\left[\begin{array}{cccc} \theta \left(a_{1}^{v}(1),a_{2}^{v}(1)\right) & \theta \left(a_{1}^{v}(2),a_{2}^{v}(2)\right) & \cdots & \theta \left(a_{1}^{v}(M),a_{2}^{v}(M)\right)\\ \theta \left(a_{1}^{v}(1),a_{3}^{v}(1)\right) & \theta \left(a_{1}^{v}(2),a_{3}^{v}(2)\right) & \cdots & \theta \left(a_{1}^{v}(M),a_{3}^{v}(M)\right)\\ \vdots & \vdots & \ddots & \vdots \\ \theta \left(a_{1}^{v}(1),a_{K}^{v}(1)\right) & \theta \left(a_{1}^{v}(2),a_{K}^{v}(2)\right) & \cdots & \theta \left(a_{1}^{v}(M),a_{K}^{v}(M)\right)\\ \theta \left(a_{2}^{v}(1),a_{3}^{v}(1)\right) & \theta \left(a_{2}^{v}(2),a_{3}^{v}(2)\right) & \cdots & \theta \left(a_{2}^{v}(M),a_{3}^{v}(M)\right)\\ \theta \left(a_{2}^{v}(1),a_{4}^{v}(1)\right) & \theta \left(a_{2}^{v}(2),a_{4}^{v}(2)\right) & \cdots & \theta \left(a_{2}^{v}(M),a_{4}^{v}(M)\right)\\ \vdots & \vdots & \ddots & \vdots \\ \theta \left(a_{2}^{v}(1),a_{K}^{v}(1)\right) & \theta \left(a_{2}^{v}(2),a_{K}^{v}(2)\right) & \cdots & \theta \left(a_{2}^{v}(M),a_{K}^{v}(M)\right)\\ \vdots & \vdots & \ddots & \vdots \\ \theta \left(a_{K-1}^{v}(1),a_{K}^{v}(1)\right) & \theta \left(a_{K-1}^{v}(2),a_{K}^{v}(2)\right) & \cdots & \theta \left(a_{K-1}^{v}(M),a_{K}^{v}(M)\right) \end{array}\right]}_{\frac{K(K-1)}{2}\times M}$$

**Step 3.** Establish a nonlinear optimization model to obtain customer evaluation information aggregation factor.

In the FGRA method, the grey relational degree is a quantitative index measuring the relationship of different sequences. To obtain the relationship between $C_{k}$ and $C_{j}$ with respect to the evaluation sequence relationship of $CR_{m}$, the grey correlation degree between the evaluation sequence of $C_{k}$ and $C_{j}$ is calculated to determine the customer weight. The grey correlation degree is obtained by using Equation (30):

(30) $$y(CAS_{k},CAS_{j})=\sum_{m=1}^{M}\omega_{m}\theta \left(a_{k}^{v}(m),a_{j}^{v}(m)\right)$$

In this expression, $w_{m}$ is customer evaluation information aggregation factor, $\sum_{m=1}^{M}w_{m}=1,0\leq w_{m}\leq 1$. The variable $w_{m}$ depends on the decision-making issue itself or is determined by the decision-maker. Therefore, establishing how to determine $w_{m}$ is critical for calculating the grey correlation coefficient between the evaluation sequence of $C_{k}$ and $C_{j}$. Based on the matrix *R*, a nonlinear optimization model can be constricted to obtain the customer evaluation information aggregation factor.

(31) $$\begin{array}{l} \min f(w)=\left|RW\right|=\sqrt{{\left(RW\right)}^{T}\cdot (RW)}\\ s.t\{\begin{array}{c} \sum_{m=1}^{M}w_{m}=1\\ 0\leq w_{m}\leq 1 \end{array} \end{array}$$

where $W={\left(w_{1},w_{2},w_{m},\ldots ,w_{M}\right)}^{T}$ is the vector of customer evaluation information aggregate factor; and *w_m_* represents the solution variables. When the gray correlation coefficient of $C_{k}$ and $C_{j}$ regarding the customer requirement $CR_{m}$ is larger, the difference of their evaluation information about $CR_{m}$ is smaller; and the contribution of $CR_{m}$ in distinguishing the evaluation sequences of $C_{k}$ and $C_{j}$ is smaller. Therefore, the minimum objective function can ensure that the corresponding information aggregation factor of $CR_{m}$ is smaller. At the same time, the information aggregation factor corresponding to the $CR_{m}$, with a small grey correlation coefficient, can take a larger value. This ensures that the $CR_{m}$ plays a greater role in distinguishing the relationships between different CAS.

**Step 4.** Establish the customer weights.

Based on $W={\left(w_{1},w_{2},w_{m},\ldots ,w_{M}\right)}^{T}$, we can calculate the grey relational degree of $CAS_{k}$ and $CAS_{j}$. The gray correlation degree is recorded as $c_{kj}$. The customer evolution consistency matrix *C* is constructed as:

(32) $$C=\left[\begin{array}{cccc} c_{11} & c_{12} & \cdots & c_{1k}\\ c_{21} & c_{22} & \cdots & c_{2k}\\ \vdots & \vdots & \ddots & \vdots \\ c_{k1} & c_{k2} & \cdots & c_{kk} \end{array}\right]$$

In Equation (32), $\omega =\left(\omega_{1},\omega_{2},\ldots ,\omega_{k},\ldots ,\omega_{K}\right)$ is the vector of the customer weight; the customer weight is defined as follows:

(33) $$\omega =\frac{\lambda_{k}}{\sum_{k=1}^{K}\lambda_{k}},k=1,2,\ldots ,K$$

where $\lambda_{k}=\sum_{j=1}^{K}c_{kj}$. A larger $\lambda_{k}$ indicates that the evaluation sequence of customer $C_{k}$ is closer to the evaluation sequence of other customers. As such, $C_{k}$ should be assigned a higher weight.

**Step 5.** Establish the customers’ key requirements based on customer weights.

Based on the calculated weight of customers, the relative importance score of all customers is obtained using Equation (34):

(34) $$IS_{m}=\sum_{k=1}^{K}\omega_{k}a_{k}^{v}(m),m=1,2,\ldots ,M$$

Finally, a ranking of $CR_{m}$ is established based on the size of $IS_{m}$.

## 4. Case Study

Because perceiving key requirements of customer collaboration low-carbon product design is a very complicated process, especially determining the weight of customers and obtaining customer evaluation information aggregation factor, we have to use numerical examples to prove the effectiveness and scientificity of the proposed method.

A company plans to develop a low carbon liquid crystal display, according to the method in [62]. Fourteen low carbon requirements were determined, each clearly affecting carbon emissions. Table 5 lists these requirements. Based on customer purchases, we randomly selected five ordinary customers, five creative customers and five leading customers. We used interval numbers, triangular fuzzy numbers and trapezoidal fuzzy numbers to describe the evaluation information of ordinary customers, creative customers and leading customers. An evaluation language with seven labels was adopted: that is s = (Very unimportant (VU); unimportant (U); Less important (LI); General important (GI); More important (MI); Important (I); Very important (VI)), as is shown in Table A1. Table A2, Table A3 and Table A4 show the results. Table A5 shows the language semantics of customer language evaluation information using different fuzzy numbers. The method proposed in this study was applied to determine the key low carbon requirements that customers have with respect to the liquid crystal display.

**Step 1.** According to the evaluation language information of customers and the corresponding fuzzy numbers, the evaluation information is converted. The conversion result is shown in Table A6, Table A7 and Table A8.

There are different uncertainties associated with customers in evaluating low carbon requirement evaluations. As such, the form of evaluation information varies. To compare all customer evaluation information, standardized different forms evaluation information is necessary. According to the interval valued definition, the result of the standardization is shown in Table A9.

**Step 2.** The calculation of grey self-correlation coefficients.

During the FGRA process, when the reference sequence is known, a grey correlation coefficient matrix can be constructed. In contrast, when the reference sequence is unknown, a grey self-correlation coefficient matrix should be built. Considering the diversification of the customer evaluation sequence, when constructing grey self-correlation coefficient matrix, we should first establish a grey relational difference information space. Then, the grey correlation coefficient is calculated. The grey self-correlation coefficient is shown in Table A10.

**Step 3.** A nonlinear optimization model is proposed to establish the information aggregation factor of customers.

**Figure 8:**
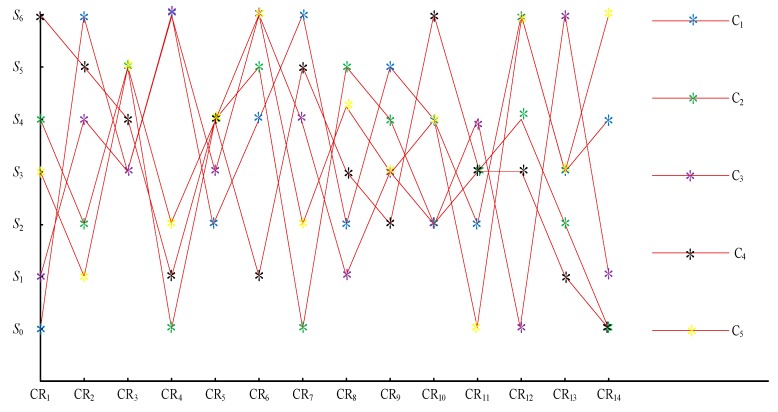
Customer evaluation language (C1–C5).

Different customers have different evaluations on a specific requirement, which is shown as an example in Figure 8. The picture shows a significant difference in the evaluation language for different customers. Thus, the different requirements should be different.

**Figure 9:**
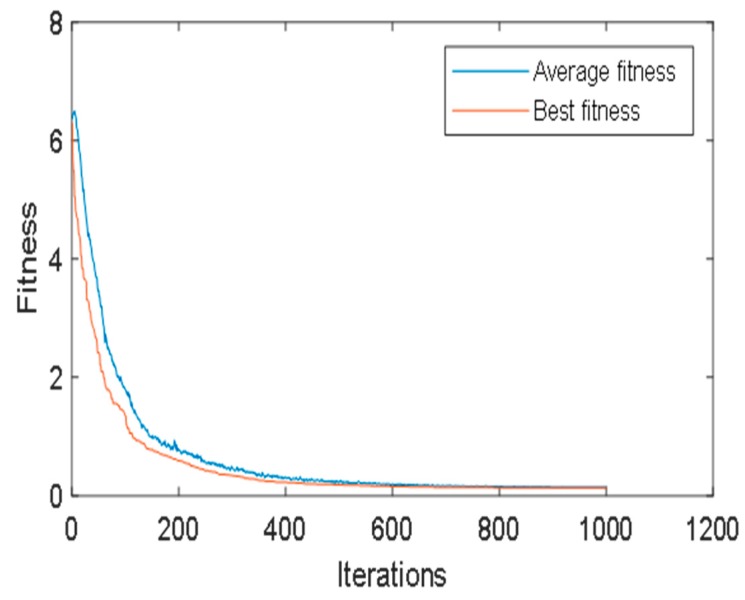
The iteration process.

Based on grey self-correlation coefficients, we present a nonlinear optimization model. Then, genetic algorithm is used to solve the model. The algorithm is coded on Matlab platform and run on Pentium 4, 2.8 GHz clock pulse with 512 MB memory. It is run 10 times using the following parameters: population size = 300; crossover rate = 0.6, mutation rate = 0.1; and number of generation = 500. These parameters have been determined after preliminary experiments. Finally, the vector of customer evaluation information aggregation factor W is obtained: W = (0.0012, 0.0008, 0.0011, 0.0011, 0.0008, 0.0012, 0.0009, 0.0012, 0.0022, 0.0008, 0.0003, 0.0014, 0.0010, 0.0006). Figure 9 shows the iteration.

**Step 4.** Establish the weight of customers.

Different consumers have different educational backgrounds, product experiences, professional knowledge, and judgment abilities. This leads to a significant degree of uncertainty in evaluating product information. It is necessary to determine the weight of the customer. Based on the results of Step 3, we obtain a consistency matrix for the customer evaluation.

$$C=\left[\begin{array}{ccccccccccccccc} 0.015 & 0.008 & 0.009 & 0.008 & 0.009 & 0.008 & 0.008 & 0.009 & 0.009 & 0.009 & 0.009 & 0.008 & 0.009 & 0.009 & 0.008\\ 0.008 & 0.015 & 0.008 & 0.008 & 0.010 & 0.010 & 0.008 & 0.009 & 0.009 & 0.009 & 0.009 & 0.009 & 0.008 & 0.008 & 0.009\\ 0.009 & 0.008 & 0.015 & 0.009 & 0.009 & 0.009 & 0.007 & 0.010 & 0.010 & 0.010 & 0.010 & 0.008 & 0.011 & 0.009 & 0.008\\ 0.008 & 0.008 & 0.009 & 0.015 & 0.009 & 0.009 & 0.009 & 0.010 & 0.009 & 0.008 & 0.008 & 0.011 & 0.008 & 0.009 & 0.008\\ 0.009 & 0.010 & 0.009 & 0.009 & 0.015 & 0.010 & 0.009 & 0.010 & 0.008 & 0.008 & 0.008 & 0.010 & 0.009 & 0.008 & 0.009\\ 0.008 & 0.010 & 0.009 & 0.009 & 0.010 & 0.015 & 0.009 & 0.011 & 0.010 & 0.009 & 0.008 & 0.010 & 0.010 & 0.009 & 0.010\\ 0.008 & 0.008 & 0.007 & 0.009 & 0.009 & 0.009 & 0.015 & 0.007 & 0.009 & 0.007 & 0.008 & 0.009 & 0.009 & 0.008 & 0.011\\ 0.009 & 0.009 & 0.010 & 0.010 & 0.010 & 0.011 & 0.007 & 0.015 & 0.009 & 0.011 & 0.008 & 0.010 & 0.010 & 0.009 & 0.009\\ 0.009 & 0.009 & 0.010 & 0.009 & 0.008 & 0.010 & 0.009 & 0.009 & 0.015 & 0.008 & 0.010 & 0.008 & 0.010 & 0.009 & 0.009\\ 0.009 & 0.009 & 0.010 & 0.008 & 0.008 & 0.009 & 0.007 & 0.011 & 0.008 & 0.015 & 0.010 & 0.009 & 0.009 & 0.010 & 0.009\\ 0.009 & 0.009 & 0.010 & 0.008 & 0.008 & 0.008 & 0.008 & 0.008 & 0.010 & 0.010 & 0.015 & 0.008 & 0.010 & 0.011 & 0.008\\ 0.008 & 0.009 & 0.008 & 0.011 & 0.010 & 0.010 & 0.009 & 0.010 & 0.008 & 0.009 & 0.008 & 0.015 & 0.009 & 0.009 & 0.010\\ 0.009 & 0.008 & 0.011 & 0.008 & 0.009 & 0.010 & 0.009 & 0.010 & 0.010 & 0.009 & 0.010 & 0.009 & 0.015 & 0.008 & 0.010\\ 0.009 & 0.008 & 0.009 & 0.009 & 0.008 & 0.009 & 0.008 & 0.009 & 0.009 & 0.010 & 0.011 & 0.009 & 0.008 & 0.015 & 0.007\\ 0.008 & 0.009 & 0.008 & 0.008 & 0.009 & 0.010 & 0.011 & 0.009 & 0.009 & 0.009 & 0.008 & 0.010 & 0.010 & 0.007 & 0.015 \end{array}\right]$$

Finally, the vector of customer’ weight ω is obtained by Equation (33): $\omega =\left[\begin{array}{ccccccccccccccc} 0.0644 & 0.0647 & 0.0669 & 0.0661 & 0.0671 & 0.0690 & 0.0634 & 0.0692 & 0.0669 & 0.0669 & 0.0660 & 0.0679 & 0.0689 & 0.0659 & 0.0667 \end{array}\right]$.

**Step 5.** Determine the key low carbon requirements of customers.

Based on the calculated weight of customers, the relative importance score of all customers is obtained using Equation (34).The result of calculated importance score of customer requirements is shown in Table 6 according to customer weights.

In Table 6, higher importance scores indicate that the customer requirement is more important. The result of these customer requirements is $CR_{12}≻CR_{6}≻CR_{10}≻CR_{13}≻CR_{3}≻CR_{4}≻CR_{7}\approx CR_{9}≻CR_{1}≻CR_{8}≻CR_{5}≻CR_{2}≻CR_{14}≻CR_{11}$.

Due to the resource restriction, it is impossible to consider all requirements in the process of customer collaborative design of low carbon products. Therefore, we identified five requirements for which the important score is higher as the key customers of low carbon: using low carbon raw materials, improving the recyclability of material, reducing the consumption of raw materials, reducing the consumption of using, and using clean energy.

## 5. Discussion

Based on the calculations above, Table 6 shows the importance score of each low carbon requirement. Based on FGRA, the low carbon requirements were sorted according to the size of important score: $CR_{12}≻CR_{6}≻CR_{10}≻CR_{13}≻CR_{3}≻CR_{4}≻CR_{7}\approx CR_{9}≻CR_{1}≻CR_{8}≻CR_{5}≻CR_{2}≻CR_{14}≻CR_{11}$. Hence, using low carbon raw materials, improving the recyclability of material, reducing the consumption of raw materials, reducing the consumption of using and using clean energy are the key low carbon requirements of customers. To verify the validity of the developed method, the Technique for Order of Preference by Similarity to an Ideal Solution (TOPSIS) [65] and the VlseKriterjumska Optimizacija I KompromisnoResenje (VIKOR) [66,67] were compared with the methods proposed in this study. The results of the comparison are shown in Table 7.

The evaluation results in Table 7 and Table 8 show slight differences in the sorting results of the three different methods. The result of the proposed FGRA method is $CR_{12}≻CR_{6}≻CR_{10}≻CR_{13}≻CR_{3}≻CR_{4}≻CR_{7}\approx CR_{9}≻CR_{1}≻CR_{8}≻CR_{5}≻CR_{2}≻CR_{14}≻CR_{11}$; the result obtained by the method of TOPSIS is $CR_{12}≻CR_{6}≻CR_{13}≻CR_{3}≻CR_{10}≻CR_{4}≻CR_{7}≻CR_{8}≻CR_{1}≻CR_{9}≻CR_{5}≻CR_{2}≻CR_{11}≻CR_{14}$; and the result of VIKOR method is $CR_{12}≻CR_{6}≻CR_{10}≻CR_{3}≻CR_{13}≻CR_{4}≻CR_{1}≻CR_{7}≻CR_{5}≻CR_{9}≻CR_{8}≻CR_{2}≻CR_{14}≻CR_{11}$. However, this does not affect the correctness of identifying the key low carbon requirements of customers. The VIKOR and TOPSIS method have some limitations in the determination of weight information. In addition, the TOPSIS method relies solely on the data itself and is prone to reverse the phenomenon, so it is very appropriate to perceiving key requirements of customer with the FGRA. FGRA method is obtained based on the combination of fuzzy number and grey relational analysis, which fully considered the fuzziness and uncertainty of the customer evaluation language. The result of discussion and comparative analysis is closer to the actual situation and can better guide the production activities of enterprises. At the same time, it demonstrates the effectiveness and feasibility of the proposed method.

## 6. Conclusions

In this paper, fuzzy grey relational analysis and genetic algorithm were combined to determine key low-carbon requirements of customers. The following conclusions were drawn.

(1) The fuzzy grey relational analysis method which considers the relationship among low carbon requirements and the relationship among customers is used in the field of customer collaborative products innovation.
(2) The hybrid fuzzy number represents the evaluation information of customers and could improve the accuracy of low carbon product in CCPI process.
(3) In this study, we introduced genetic algorithm into fuzzy grey relational analysis, and proposed a method for standardizing fuzzy information in an uncertain environment. We also constructed a nonlinear optimization model, solving the realistic problem that customer weight is not easily determined.
(4) Customer collaborative innovation of low-carbon products is an important trend. Determinations of key low-carbon requirements of customers can enhance enterprise competitiveness, reduce carbon emissions and protect the environment.

This paper considers the heterogeneity of customers and uses different fuzzy numbers to describe their evaluation language, but does not consider the customer’s psychological behavior factors. The psychological behavioral factors of customers have a certain influence on the accuracy of the perceiving key requirements of customer collaboration low-carbon product design. The size of the specific influence is the direction of future research.

## Figures and Tables

**Table 1:** The relationship of language semantics and interval numbers.

Language Labels	Language Semantics	Interval Numbers
Five labels	*S*_0_	(0.000, 0.200)
	*S*_1_	(0.200, 0.400)
	*S*_2_	(0.400, 0.600)
	*S*_3_	(0.600, 0.800)
	*S*_4_	(0.800, 1.000)
Seven labels	*S*_0_	(0.000, 0.143)
	*S*_1_	(0.143, 0.285)
	*S*_2_	(0.285, 0.428)
	*S*_3_	(0.428, 0.572)
	*S*_4_	(0.572, 0.715)
	*S*_5_	(0.715, 0.857)
	*S*_6_	(0.857, 1.000)
Nine labels	*S*_0_	(0.000, 0.111)
	*S*_1_	(0.111, 0.222)
	*S*_2_	(0.222, 0.333)
	*S*_3_	(0.333, 0.444)
	*S*_4_	(0.444, 0.555)
	*S*_5_	(0.555, 0.666)
	*S*_6_	(0.666, 0.777)
	*S*_7_	(0.777, 0.888)
	*S*_8_	(0.888, 1.000)

**Table 2:** The relationship between language semantics and the triangular fuzzy numbers.

Language Labels	Language Semantics	Triangular Fuzzy Numbers
Five labels	*S*_0_	(0.000, 0.000, 0.250)
	*S*_1_	(0.000, 0.250, 0.500)
	*S*_2_	(0.250, 0.500, 0.750)
	*S*_3_	(0.500, 0.750, 1.000)
	*S*_4_	(0.750, 1.000, 1.000)
Seven labels	*S*_0_	(0.000, 0.000, 0.166)
	*S*_1_	(0.000, 0.166, 0.333)
	*S*_2_	(0.166, 0.333, 0.499)
	*S*_3_	(0.333, 0.499, 0.667)
	*S*_4_	(0.499, 0.667, 0.833)
	*S*_5_	(0.667, 0.833, 1.000)
	*S*_6_	(0.833, 1.000, 1.000)
Nine labels	*S*_0_	(0.000, 0.000, 0.125)
	*S*_1_	(0.000, 0.125, 0.250)
	*S*_2_	(0.125, 0.250, 0.375)
	*S*_3_	(0.250, 0.375, 0.500)
	*S*_4_	(0.375, 0.500, 0.625)
	*S*_5_	(0.500, 0.625, 0.750)
	*S*_6_	(0.625, 0.750, 0.875)
	*S*_7_	(0.750, 0.875, 1.000)
	*S*_8_	(0.875, 1.000, 1.000)

**Table 3:** The relationship between language semantics and trapezoidal fuzzy numbers.

Language Labels	Language Semantics	Trapezoidal Fuzzy Numbers
Five labels	*S*_0_	(0.000, 0.000, 0.111, 0.222)
	*S*_1_	(0.111, 0.222, 0.333, 0.444)
	*S*_2_	(0.333, 0.444, 0.555, 0.666)
	*S*_3_	(0.555, 0.666, 0.777, 0.888)
	*S*_4_	(0.777, 0.888, 1.000, 1.000)
Seven labels	*S*_0_	(0.000, 0.000, 0.077, 0.154)
	*S*_1_	(0.077, 0.154, 0.231, 0.308)
	*S*_2_	(0.231, 0.308, 0.385, 0.462)
	*S*_3_	(0.385, 0.462, 0.538, 0.615)
	*S*_4_	(0.538, 0.615, 0.692, 0.769)
	*S*_5_	(0.692, 0.769, 0.846, 0.923)
	*S*_6_	(0.846, 0.923, 1.000, 1.000)
Nine labels	*S*_0_	(0.000, 0.000, 0.058, 0.117)
	*S*_1_	(0.058, 0.117, 0.176, 0.235)
	*S*_2_	(0.176, 0.235, 0.294, 0.353)
	*S*_3_	(0.294, 0.353, 0.411, 0.470)
	*S*_4_	(0.411, 0.470, 0.529, 0.580)
	*S*_5_	(0.529, 0.580, 0.647, 0.706)
	*S*_6_	(0.647, 0.706, 0.765, 0.823)
	*S*_7_	(0.765, 0.823, 0.882, 0.941)
	*S*_8_	(0.882, 0.941, 1.000, 1.000)

**Table 4:** The expression of customer evaluation language.

Customer Requirements	Interval Number	Triangular Fuzzy Number	Trapezoidal Fuzzy Number
CR_1_	$a_{k}\left(1\right)=a_{k}^{I}\left(1\right)$	$a_{k}\left(1\right)=a_{k}^{S}\left(1\right)$	$a_{k}\left(1\right)=a_{k}^{T}\left(1\right)$
CR_2_	$a_{k}\left(2\right)=a_{k}^{I}\left(2\right)$	$a_{k}\left(2\right)=a_{k}^{S}\left(2\right)$	$a_{k}\left(2\right)=a_{k}^{T}\left(2\right)$
…	…	…	…
CR*_m_*	$a_{k}\left(m\right)=a_{k}^{I}\left(m\right)$	$a_{k}\left(m\right)=a_{k}^{S}\left(m\right)$	$a_{k}\left(m\right)=a_{k}^{T}\left(m\right)$

Note: $a_{k}^{I}(m)$ indicates that the evaluation results of customer *k* on the requirement *m* is an interval number. $a_{k}^{S}\left(m\right)$ shows the evaluation results of customer *k* on the requirement *m* is a triangular fuzzy number. $a_{k}^{T}\left(m\right)$ expresses the evaluation results of customer *k* on the requirement *m* is a trapezoidal fuzzy number.

**Table 5:** Low carbon requirements for customers.

Customer Requirements	Explanation
CR_1_	Reduce the consumption of design
CR_2_	Reduce the consumption of handling resource
CR_3_	Use clean energy
CR_4_	Reduce the consumption of manufacturing resource
CR_5_	Increase the rate of cooling
CR_6_	Improve the recyclability of material
CR_7_	Reduce the consumption of maintenance
CR_8_	Reduce the consumption of dismantling
CR_9_	Reduce the waste of product
CR_10_	Reduce the consumption of raw materials
CR_11_	Reduce the emission of transport
CR_12_	Use low carbon raw materials
CR_13_	Reduce the consumption of using
CR_14_	Reduce the consumption of packaging materials

**Table 6:** Importance score of low carbon requirements.

CR_1_	CR_2_	CR_3_	CR_4_	CR_5_	CR_6_	CR_7_	CR_8_	CR_9_	CR_10_	CR_11_	CR_12_	CR_13_	CR_14_
0.181	0.157	0.210	0.204	0.165	0.224	0.186	0.174	0.186	0.212	0.134	0.238	0.211	0.135

**Table 7:** Comparison result of the proposed method with the TOPSIS and the VIKOR.

Method	The Results of Methods
FGRA	$CR_{12}≻CR_{6}≻CR_{10}≻CR_{13}≻CR_{3}≻CR_{4}≻CR_{7}\approx CR_{9}≻CR_{1}≻CR_{8}≻CR_{5}≻CR_{2}≻CR_{14}≻CR_{11}$
TOPSIS	$CR_{12}≻CR_{6}≻CR_{13}≻CR_{3}≻CR_{10}≻CR_{4}≻CR_{7}≻CR_{8}≻CR_{1}≻CR_{9}≻CR_{5}≻CR_{2}≻CR_{11}≻CR_{14}$
VIKOR	$CR_{12}≻CR_{6}≻CR_{10}≻CR_{3}≻CR_{13}≻CR_{4}≻CR_{1}≻CR_{7}≻CR_{5}≻CR_{9}≻CR_{8}≻CR_{2}≻CR_{14}≻CR_{11}$

**Table 8:** Comparison result of the proposed method with the TOPSIS and the VIKOR about key low carbon requirement of customers.

Method	Key Low Carbon Requirement of Customers
FGRA	$\begin{array}{ccccc} CR_{12} & CR_{6} & CR_{10} & CR_{13} & CR_{3} \end{array}$
TOPSIS	$\begin{array}{ccccc} CR_{12} & CR_{6} & CR_{13} & CR_{3} & CR_{10} \end{array}$
VIKOR	$\begin{array}{ccccc} CR_{12} & CR_{6} & CR_{10} & CR_{3} & CR_{13} \end{array}$

## Appendix A

**Table A1:** Linguistic variables for rating relationships and assessments with respect to different criteria.

Linguistic Variable	Very Unimportant	Unimportant	Less Important	General Important	More Important	Important	Very Important
logogram	VU	U	LI	GI	MI	I	VI

**Table A2:** Ordinary customer assessment language for low carbon requirements.

Ordinary Customers	Customer Requirements													
	CR_1_	CR_2_	CR_3_	CR_4_	CR_5_	CR_6_	CR_7_	CR_8_	CR_9_	CR_10_	CR_11_	CR_12_	CR_13_	CR_14_
C_1_	VU	VI	GI	VI	LI	MI	VI	LI	I	MI	LI	VI	GI	MI
C_2_	MI	LI	I	VU	MI	I	VU	I	MI	LI	GI	MI	LI	VU
C_3_	U	MI	GI	VI	GI	VI	MI	U	GI	LI	MI	VU	VI	U
C_4_	VI	I	MI	U	MI	U	I	GI	LI	VI	GI	GI	U	VU
C_5_	GI	U	I	LI	MI	VI	LI	MI	GI	MI	VU	VI	GI	VI

**Table A3:** Creative customer assessment language for low carbon requirements.

Creative Customers	Customer Requirements													
	CR_1_	CR_2_	CR_3_	CR_4_	CR_5_	CR_6_	CR_7_	CR_8_	CR_9_	CR_10_	CR_11_	CR_12_	CR_13_	CR_14_
C_6_	MI	VU	LI	VU	I	GI	MI	VI	GI	MI	MI	MI	I	U
C_7_	VI	MI	VU	I	VI	U	GI	VI	U	VI	VU	VI	GI	VI
C_8_	VU	GI	VI	U	MI	MI	I	GI	GI	MI	VI	LI	I	VU
C_9_	I	VI	U	I	MI	GI	LI	LI	MI	VU	U	MI	VI	LI
C_10_	U	MI	VI	VU	U	MI	GI	U	VI	GI	I	VU	MI	VU

**Table A4:** Leading customer assessment language for low carbon requirements.

Leading Customers	Customer Requirements													
	CR_1_	CR_2_	CR_3_	CR_4_	CR_5_	CR_6_	CR_7_	CR_8_	CR_9_	CR_10_	CR_11_	CR_12_	CR_13_	CR_14_
C_1__1_	I	VU	LI	VI	LI	VI	VU	U	VI	LI	U	GI	U	GI
C_1__2_	GI	U	I	VU	U	VU	MI	GI	VU	I	GI	MI	LI	MI
C_1__3_	U	LI	VU	VI	U	I	LI	U	LI	MI	VU	I	VI	U
C_1__4_	GI	GI	VU	GI	MI	VU	VI	LI	VI	LI	MI	GI	VU	VI
C_1__5_	MI	LI	VI	I	LI	MI	GI	VI	VU	VI	LI	VI	I	U

**Table A5:** Language semantics of evaluation information with different fuzzy numbers.

Fuzzy Numbers	Evaluation Language	Language Semantics
Seven labels (Interval number)	*S*_0_	(0.000, 0.143)
	*S*_1_	(0.143, 0.285)
	*S*_2_	(0.285, 0.428)
	*S*_3_	(0.428, 0.572)
	*S*_4_	(0.572, 0.715)
	*S*_5_	(0.715, 0.857)
	*S*_6_	(0.857, 1.000)
Seven labels (triangular fuzzy number)	*S*_0_	(0.000, 0.000, 0.166)
	*S*_1_	(0.000, 0.166, 0.333)
	*S*_2_	(0.166, 0.333, 0.499)
	*S*_3_	(0.333, 0.499, 0.667)
	*S*_4_	(0.499, 0.667, 0.833)
	*S*_5_	(0.667, 0.833, 1.000)
	*S*_6_	(0.833, 1.000, 1.000)
Seven labels (trapezoidal fuzzy number)	*S*_0_	(0.000, 0.000, 0.077, 0.154)
	*S*_1_	(0.077, 0.154, 0.231, 0.308)
	*S*_2_	(0.231, 0.308, 0.385, 0.462)
	*S*_3_	(0.385, 0.462, 0.538, 0.615)
	*S*_4_	(0.538, 0.615, 0.692, 0.769)
	*S*_5_	(0.692, 0.769, 0.846, 0.923)
	*S*_6_	(0.846, 0.923, 1.000, 1.000)

**Table A6:** Ordinary customer assessment information.

Customer Requirements	Ordinary Customers				
	C_1_	C_2_	C_3_	C_4_	C_5_
CR_1_	(0.000, 0.143)	(0.572, 0.715)	(0.143, 0.285)	(0.857, 1.000)	(0.428, 0.572)
CR_2_	(0.857, 1.000)	(0.285, 0.428)	(0.572, 0.715)	(0.715, 0.857)	(0.143, 0.285)
CR_3_	(0.428, 0.572)	(0.715, 0.857)	(0.428, 0.572)	(0.572, 0.715)	(0.715, 0.857)
CR_4_	(0.857, 1.000)	(0.000, 0.143)	(0.857, 1.000)	(0.143, 0.285)	(0.285, 0.428)
CR_5_	(0.285, 0.428)	(0.572, 0.715)	(0.428, 0.572)	(0.572, 0.715)	(0.572, 0.715)
CR_6_	(0.572, 0.715)	(0.715, 0.857)	(0.857, 1.000)	(0.143, 0.285)	(0.857, 1.000)
CR_7_	(0.857, 1.000)	(0.000, 0.143)	(0.572, 0.715)	(0.715, 0.857)	(0.285, 0.428)
CR_8_	(0.285, 0.428)	(0.715, 0.857)	(0.143, 0.285)	(0.428, 0.572)	(0.572, 0.715)
CR_9_	(0.715, 0.857)	(0.572, 0.715)	(0.428, 0.572)	(0.285, 0.428)	(0.428, 0.572)
CR_10_	(0.572, 0.715)	(0.285, 0.428)	(0.285, 0.428)	(0.857, 1.000)	(0.572, 0.715)
CR_11_	(0.285, 0.428)	(0.428, 0.572)	(0.572, 0.715)	(0.428, 0.572)	(0.000, 0.143)
CR_12_	(0.857, 1.000)	(0.572, 0.715)	(0.000, 0.143)	(0.428, 0.572)	(0.857, 1.000)
CR_13_	(0.428, 0.572)	(0.285, 0.428)	(0.857, 1.000)	(0.143, 0.285)	(0.428, 0.572)
CR_14_	(0.572, 0.715)	(0.000, 0.143)	(0.143, 0.285)	(0.000, 0.143)	(0.857, 1.000)

**Table A7:** Creative customer assessment information.

Customer Requirements	Creative Customers				
	C_6_	C_7_	C_8_	C_9_	C_10_
CR_1_	(0.499, 0.667, 0.833)	(0.833, 1.000, 1.000)	(0.000, 0.000, 0.166)	(0.667, 0.833, 1.000)	(0.000, 0.166, 0.333)
CR_2_	(0.000, 0.000, 0.166)	(0.499, 0.667, 0.833)	(0.333, 0.499, 0.667)	(0.833, 1.000, 1.000)	(0.499, 0.667, 0.833)
CR_3_	(0.166, 0.333, 0.499)	(0.000, 0.000, 0.166)	(0.833, 1.000, 1.000)	(0.000, 0.166, 0.333)	(0.833, 1.000, 1.000)
CR_4_	(0.000, 0.000, 0.166)	(0.667, 0.833, 1.000)	(0.000, 0.166, 0.333)	(0.667, 0.833, 1.000)	(0.000, 0.000, 0.166)
CR_5_	(0.667, 0.833, 1.000)	(0.833, 1.000, 1.000)	(0.499, 0.667, 0.833)	(0.499, 0.667, 0.833)	(0.000, 0.166, 0.333)
CR_6_	(0.333, 0.499, 0.667)	(0.000, 0.166, 0.333)	(0.499, 0.667, 0.833)	(0.333, 0.499, 0.667)	(0.499, 0.667, 0.833)
CR_7_	(0.499, 0.667, 0.833)	(0.333, 0.499, 0.667)	(0.667, 0.833, 1.000)	(0.166, 0.333, 0.499)	(0.333, 0.499, 0.667)
CR_8_	(0.833, 1.000, 1.000)	(0.833, 1.000, 1.000)	(0.333, 0.499, 0.667)	(0.166, 0.333, 0.499)	(0.000, 0.166, 0.333)
CR_9_	(0.333, 0.499, 0.667)	(0.000, 0.166, 0.333)	(0.333, 0.499, 0.667)	(0.499, 0.667, 0.833)	(0.833, 1.000, 1.000)
CR_10_	(0.499, 0.667, 0.833)	(0.833, 1.000, 1.000)	(0.499, 0.667, 0.833)	(0.000, 0.000, 0.166)	(0.333, 0.499, 0.667)
CR_11_	(0.499, 0.667, 0.833)	(0.000, 0.000, 0.166)	(0.833, 1.000, 1.000)	(0.000, 0.166, 0.333)	(0.667, 0.833, 1.000)
CR_12_	(0.499, 0.667, 0.833)	(0.833, 1.000, 1.000)	(0.166, 0.333, 0.499)	(0.499, 0.667, 0.833)	(0.000, 0.000, 0.166)
CR_13_	(0.667, 0.833, 1.000)	(0.333, 0.499, 0.667)	(0.667, 0.833, 1.000)	(0.833, 1.000, 1.000)	(0.499, 0.667, 0.833)
CR_14_	(0.000, 0.166, 0.333)	(0.833, 1.000, 1.000)	(0.000, 0.000, 0.166)	(0.166, 0.333, 0.499)	(0.000, 0.000, 0.166)

**Table A8:** Leading customer assessment information.

Customer Requirements	Leading Customers				
	C_11_	C_12_	C_13_	C_14_	C_15_
CR_1_	(0.692, 0.769, 0.846, 0.923)	(0.385, 0.462, 0.538, 0.615)	(0.077, 0.154, 0.231, 0.308)	(0.385, 0.462, 0.538, 0.615)	(0.538, 0.615, 0.692, 0.769)
CR_2_	(0.000, 0.000, 0.077, 0.154)	(0.077, 0.154, 0.231, 0.308)	(0.231, 0.308, 0.385, 0.462)	(0.385, 0.462, 0.538, 0.615)	(0.231, 0.308, 0.385, 0.462)
CR_3_	(0.231, 0.308, 0.385, 0.462)	(0.692, 0.769, 0.846, 0.923)	(0.000, 0.000, 0.077, 0.154)	(0.000, 0.000, 0.077, 0.154)	(0.846, 0.923, 1.000, 1.000)
CR_4_	(0.846, 0.923, 1.000, 1.000)	(0.000, 0.000, 0.077, 0.154)	(0.846, 0.923, 1.000, 1.000)	(0.385, 0.462, 0.538, 0.615)	(0.692, 0.769, 0.846, 0.923)
CR_5_	(0.231, 0.308, 0.385, 0.462)	(0.077, 0.154, 0.231, 0.308)	(0.077, 0.154, 0.231, 0.308)	(0.538, 0.615, 0.692, 0.769)	(0.231, 0.308, 0.385, 0.462)
CR_6_	(0.846, 0.923, 1.000, 1.000)	(0.000, 0.000, 0.077, 0.154)	(0.692, 0.769, 0.846, 0.923)	(0.000, 0.000, 0.077, 0.154)	(0.538, 0.615, 0.692, 0.769)
CR_7_	(0.000, 0.000, 0.077, 0.154)	(0.538, 0.615, 0.692, 0.769)	(0.231, 0.308, 0.385, 0.462)	(0.846, 0.923, 1.000, 1.000)	(0.385, 0.462, 0.538, 0.615)
CR_8_	(0.077, 0.154, 0.231, 0.308)	(0.385, 0.462, 0.538, 0.615)	(0.077, 0.154, 0.231, 0.308)	(0.231, 0.308, 0.385, 0.462)	(0.846, 0.923, 1.000, 1.000)
CR_9_	(0.846, 0.923, 1.000, 1.000)	(0.000, 0.000, 0.077, 0.154)	(0.231, 0.308, 0.385, 0.462)	(0.846, 0.923, 1.000, 1.000)	(0.000, 0.000, 0.077, 0.154)
CR_10_	(0.231, 0.308, 0.385, 0.462)	(0.692, 0.769, 0.846, 0.923)	(0.538, 0.615, 0.692, 0.769)	(0.231, 0.308, 0.385, 0.462)	(0.846, 0.923, 1.000, 1.000)
CR_11_	(0.077, 0.154, 0.231, 0.308)	(0.385, 0.462, 0.538, 0.615)	(0.000, 0.000, 0.077, 0.154)	(0.538, 0.615, 0.692, 0.769)	(0.231, 0.308, 0.385, 0.462)
CR_12_	(0.385, 0.462, 0.538, 0.615)	(0.538, 0.615, 0.692, 0.769)	(0.692, 0.769, 0.846, 0.923)	(0.385, 0.462, 0.538, 0.615)	(0.846, 0.923, 1.000, 1.000)
CR_13_	(0.077, 0.154, 0.231, 0.308)	(0.231, 0.308, 0.385, 0.462)	(0.846, 0.923, 1.000, 1.000)	(0.000, 0.000, 0.077, 0.154)	(0.692, 0.769, 0.846, 0.923)
CR_14_	(0.385, 0.462, 0.538, 0.615)	(0.538, 0.615, 0.692, 0.769)	(0.077, 0.154, 0.231, 0.308)	(0.846, 0.923, 1.000, 1.000)	(0.077, 0.154, 0.231, 0.308)

**Table A9:** Interval value processing of customer evaluation information.

Customer Requirements	Customers														
	C_1_	C_2_	C_3_	C_4_	C_5_	C_6_	C_7_	C_8_	C_9_	C_10_	C_11_	C_12_	C_13_	C_14_	C_15_
CR_1_	0.000	0.320	0.014	0.500	0.125	0.213	0.448	0.000	0.344	0.010	0.318	0.131	0.011	0.111	0.201
CR_2_	0.500	0.080	0.223	0.348	0.014	0.000	0.213	0.115	0.448	0.213	0.000	0.013	0.048	0.111	0.048
CR_3_	0.125	0.500	0.125	0.223	0.348	0.047	0.000	0.448	0.010	0.448	0.048	0.376	0.000	0.000	0.443
CR_4_	0.500	0.000	0.500	0.014	0.055	0.000	0.344	0.010	0.344	0.000	0.443	0.000	0.443	0.111	0.318
CR_5_	0.055	0.320	0.125	0.223	0.223	0.344	0.448	0.213	0.213	0.010	0.048	0.013	0.011	0.201	0.048
CR_6_	0.223	0.500	0.500	0.014	0.500	0.115	0.010	0.213	0.115	0.213	0.443	0.000	0.318	0.000	0.201
CR_7_	0.500	0.000	0.223	0.348	0.055	0.213	0.115	0.344	0.047	0.115	0.000	0.237	0.048	0.443	0.111
CR_8_	0.055	0.500	0.014	0.125	0.223	0.448	0.448	0.115	0.047	0.010	0.011	0.131	0.011	0.048	0.443
CR_9_	0.348	0.320	0.125	0.055	0.125	0.115	0.010	0.115	0.213	0.448	0.443	0.000	0.048	0.443	0.000
CR_10_	0.223	0.080	0.055	0.500	0.223	0.213	0.448	0.213	0.000	0.115	0.048	0.376	0.201	0.048	0.443
CR_11_	0.055	0.180	0.223	0.125	0.000	0.213	0.000	0.448	0.010	0.344	0.011	0.131	0.000	0.201	0.048
CR_12_	0.500	0.320	0.000	0.125	0.500	0.213	0.448	0.047	0.213	0.000	0.111	0.237	0.318	0.111	0.443
CR_13_	0.125	0.080	0.500	0.014	0.125	0.344	0.115	0.344	0.448	0.213	0.011	0.056	0.443	0.000	0.318
CR_14_	0.223	0.000	0.014	0.000	0.500	0.010	0.448	0.000	0.047	0.000	0.111	0.237	0.011	0.443	0.011

**Table A10:** The grey self-correlation coefficients matrix *R*.

CR_1_	CR_2_	CR_3_	CR_4_	CR_5_	CR_6_	CR_7_	CR_8_	CR_9_	CR_10_	CR_11_	CR_12_	CR_13_	CR_14_
0.439	0.373	0.400	0.333	0.452	0.474	0.333	0.355	0.889	0.636	0.642	0.581	0.847	0.529
0.947	0.474	1.000	1.000	0.758	0.474	0.474	0.857	0.501	0.598	0.571	0.333	0.400	0.545
0.333	0.622	0.718	0.340	0.566	0.545	0.622	0.778	0.433	0.474	0.762	0.400	0.693	0.529
0.667	0.340	0.529	0.360	0.566	0.474	0.360	0.593	0.501	1.000	0.803	1.000	1.000	0.474
0.540	0.333	0.762	0.333	0.431	0.698	0.466	0.384	0.490	0.962	0.586	0.466	0.533	0.540
0.358	0.466	0.667	0.616	0.358	0.540	0.394	0.384	0.399	0.526	0.803	0.828	0.962	0.526
1.000	0.394	0.436	0.338	0.581	0.962	0.616	0.803	0.490	0.962	0.363	0.356	0.533	0.529
0.421	0.828	0.685	0.616	0.581	0.698	0.356	0.968	0.624	0.529	0.833	0.466	0.436	0.587
0.962	0.466	0.436	0.333	0.830	0.962	0.394	0.845	0.691	0.698	0.437	0.333	0.740	0.529
0.440	0.333	0.765	0.814	0.969	0.532	0.333	0.848	0.702	0.588	0.836	0.391	0.687	0.691
0.656	0.339	0.499	0.333	0.839	0.529	0.487	0.763	0.392	0.620	0.747	0.487	0.784	0.947
0.958	0.356	0.667	0.814	0.833	0.725	0.356	0.848	0.427	0.919	0.803	0.579	0.440	0.541
0.693	0.391	0.667	0.391	0.600	0.529	0.814	0.972	0.702	0.588	0.605	0.391	0.667	0.532
0.554	0.356	0.440	0.579	0.969	0.919	0.391	0.387	0.392	0.532	0.970	0.814	0.564	0.541
0.450	0.636	0.400	0.333	0.529	1.000	0.529	0.335	0.535	0.909	0.839	0.439	0.373	0.947
0.581	0.483	0.474	0.947	0.693	0.340	0.418	0.395	0.458	0.373	0.803	0.562	0.791	1.000
0.562	0.791	0.622	0.820	0.693	1.000	0.820	0.469	0.535	0.636	0.554	0.581	0.847	0.333
0.700	0.758	0.356	1.000	0.901	0.394	0.540	0.825	0.522	0.653	0.872	0.700	0.486	0.962
0.661	0.653	0.333	0.421	0.631	0.338	0.685	0.825	0.419	0.405	0.554	0.661	0.877	0.358
0.439	0.877	0.828	0.962	0.672	0.466	0.421	0.389	0.522	0.653	0.455	0.478	0.486	1.000
0.912	0.405	0.338	0.421	0.672	0.394	0.842	0.351	0.677	0.758	0.569	0.700	0.405	0.842
0.446	0.653	0.828	1.000	0.414	0.466	0.685	0.333	0.636	0.877	0.577	0.439	0.653	1.000
0.992	0.758	0.356	0.361	0.446	0.814	1.000	0.334	0.646	0.887	0.570	0.545	0.784	0.693
0.569	0.789	0.668	1.000	0.416	0.333	0.513	0.399	0.412	0.458	0.821	0.751	0.912	0.513
0.447	0.887	0.333	0.361	0.415	0.579	0.839	0.334	0.452	0.674	0.554	0.992	0.408	0.958
0.545	0.890	0.333	0.693	0.648	0.333	0.361	0.352	0.646	0.887	0.914	0.545	0.758	0.361
0.678	0.887	0.814	0.440	0.446	0.455	0.693	0.811	0.412	0.408	0.629	0.670	0.512	0.958
0.340	0.667	0.718	0.340	0.691	0.340	0.667	0.688	0.762	0.360	0.696	0.667	0.340	0.947
0.693	0.545	0.529	0.360	0.691	1.000	0.598	0.540	1.000	0.598	0.501	0.333	0.400	0.340
0.557	0.529	0.762	0.333	0.500	0.394	0.962	0.361	0.957	0.613	0.957	0.540	0.616	0.984
0.365	0.962	0.667	0.616	0.404	0.338	0.698	0.361	0.661	0.389	0.501	0.358	0.394	0.365
0.947	0.698	0.436	0.338	0.713	0.466	0.674	0.708	0.957	0.613	0.499	0.842	0.616	0.947
0.431	0.526	0.685	0.616	0.713	0.394	0.587	0.881	0.718	0.820	0.513	0.540	0.828	0.883
0.984	0.962	0.436	0.333	0.656	0.466	0.698	0.984	0.410	0.806	0.649	1.000	0.466	0.947
0.451	0.529	0.765	0.814	0.740	0.814	0.529	0.988	0.413	0.973	0.514	0.693	0.338	0.720
0.681	0.543	0.499	0.333	0.662	0.333	0.947	0.677	0.642	0.438	0.709	0.513	0.360	0.529
0.988	0.588	0.667	0.814	0.658	0.579	0.588	0.988	0.744	0.631	0.501	0.440	0.814	0.988
0.720	0.691	0.667	0.391	0.742	0.333	0.532	0.878	0.413	0.973	0.911	0.693	0.333	0.368
0.572	0.588	0.440	0.579	0.740	0.455	0.691	0.364	0.642	0.392	0.561	0.361	0.579	0.988
0.400	0.428	0.667	0.859	1.000	0.340	0.460	0.714	0.762	0.474	0.642	0.400	0.693	0.333
0.466	0.418	0.587	0.947	0.644	0.712	0.649	0.431	0.789	0.466	0.718	0.740	0.431	0.962
0.828	0.649	0.529	0.431	0.493	0.984	0.518	0.431	0.833	0.828	0.642	0.436	0.712	0.358
0.333	0.518	0.526	0.984	0.956	0.557	0.984	0.961	0.789	0.466	0.410	0.762	0.431	1.000
0.616	0.714	0.540	0.431	0.956	0.712	0.454	0.759	0.586	0.333	0.661	0.740	0.365	0.842
0.338	0.649	0.526	0.947	0.507	0.557	0.518	0.681	0.363	0.394	0.506	0.667	0.557	1.000
0.579	0.418	0.588	0.368	0.556	0.368	0.418	0.682	0.366	0.356	0.663	0.947	0.988	0.693
0.404	0.427	0.620	0.947	0.510	0.947	0.693	0.976	0.803	0.668	0.974	0.691	0.856	0.513
0.338	0.455	0.529	0.368	0.508	0.451	0.455	0.682	0.970	0.455	0.642	0.564	0.368	0.958
0.391	0.513	0.529	0.720	0.909	0.947	0.725	0.761	0.366	0.356	0.747	0.947	0.947	0.361
0.455	0.455	0.532	0.451	0.556	0.572	0.513	0.435	0.803	0.814	0.744	0.440	0.451	0.958
0.740	0.947	0.454	0.820	0.644	0.394	0.613	0.521	0.957	0.962	0.513	0.466	0.533	0.338
0.436	0.557	0.418	0.464	0.493	0.338	0.806	0.521	0.661	0.526	1.000	0.828	0.962	0.828
0.667	0.712	0.714	0.847	0.956	0.466	0.464	0.694	0.957	0.962	0.333	0.356	0.533	0.333
0.533	0.365	0.425	0.464	0.956	0.394	0.969	0.582	0.718	0.529	0.957	0.466	0.436	0.356
0.685	0.557	0.714	0.820	0.507	0.466	0.806	0.535	0.410	0.698	0.394	0.333	0.740	0.333
0.564	0.947	0.455	0.392	0.556	0.814	0.820	0.536	0.413	0.588	0.953	0.391	0.687	0.391
0.977	0.996	0.899	0.820	0.510	0.333	0.579	0.727	0.642	0.620	0.631	0.487	0.784	0.487
0.687	0.880	0.418	0.392	0.508	0.579	0.973	0.536	0.744	0.919	1.000	0.579	0.440	0.338
0.947	0.720	0.418	0.817	0.909	0.333	0.392	0.583	0.413	0.588	0.527	0.391	0.667	0.814
0.767	0.880	0.725	0.487	0.556	0.455	0.817	0.527	0.642	0.532	0.824	0.814	0.564	0.338
0.515	0.540	0.842	0.421	0.678	0.704	0.718	1.000	0.681	0.515	0.513	0.515	0.522	0.363
0.540	0.685	0.384	0.962	0.626	0.718	0.656	0.424	1.000	1.000	0.488	0.601	1.000	0.962
0.656	0.358	0.871	0.421	0.626	1.000	0.601	0.379	0.696	0.540	0.525	1.000	0.706	0.871
0.552	0.540	0.384	1.000	0.396	0.718	0.718	0.359	0.402	0.718	0.631	0.540	0.656	0.962
0.704	1.000	0.996	0.361	0.425	0.433	0.540	0.359	0.406	0.602	0.526	0.710	0.429	0.712
0.753	0.951	0.432	1.000	0.398	0.685	0.912	0.436	0.661	0.605	0.732	0.912	0.465	0.524
0.553	0.839	0.842	0.361	0.397	0.552	0.602	0.359	0.770	0.954	0.513	0.704	0.716	0.996
0.710	0.693	0.842	0.693	0.605	0.685	0.521	0.380	0.406	0.602	0.949	0.710	0.421	0.366
0.954	0.839	0.387	0.440	0.425	0.744	0.710	0.980	0.661	0.521	0.576	0.521	0.906	0.996
0.358	0.718	0.358	0.428	0.482	0.552	0.522	0.424	0.681	0.515	0.333	0.384	0.522	0.358
0.706	0.515	0.962	1.000	0.482	0.704	0.786	0.379	0.525	0.358	0.957	0.515	0.429	0.384
0.363	1.000	0.358	0.421	0.333	0.552	1.000	0.359	0.338	0.429	0.394	0.358	0.718	0.358
0.658	0.540	0.839	0.716	0.354	0.366	0.685	0.359	0.341	0.385	0.953	0.426	0.706	0.426
0.441	0.556	0.399	0.421	0.335	0.962	0.672	0.436	0.957	0.776	0.631	0.542	0.809	0.542
0.364	0.602	1.000	0.716	0.334	0.448	0.789	0.359	0.855	0.503	1.000	0.658	0.433	0.364
0.426	0.710	1.000	0.518	0.470	0.962	0.433	0.380	0.341	0.385	0.527	0.426	0.685	0.980
0.503	0.602	0.361	0.906	0.354	0.567	0.984	0.980	0.957	0.980	0.824	0.980	0.552	0.364
0.421	0.429	0.363	0.428	1.000	0.718	0.457	0.783	0.696	0.540	0.338	0.601	0.706	0.842
0.962	0.718	1.000	0.962	0.519	1.000	0.522	0.700	0.402	0.718	0.683	0.842	0.656	1.000
0.440	0.685	0.385	0.366	0.570	0.521	0.421	0.702	0.406	0.602	0.339	0.796	0.429	0.693
0.656	0.710	0.776	0.962	0.523	0.540	0.700	0.939	0.661	0.605	0.414	0.568	0.465	0.513
0.958	0.789	0.358	0.366	0.520	0.704	0.458	0.702	0.770	0.954	0.333	0.480	0.716	0.958
0.693	0.984	0.358	0.712	0.948	0.540	0.716	0.785	0.406	0.602	0.476	0.796	0.421	0.361
0.554	0.789	0.980	0.448	0.570	0.954	0.518	0.428	0.661	0.521	0.359	0.387	0.906	0.958
0.428	0.515	0.363	0.421	0.519	0.718	0.786	0.869	0.488	0.685	0.401	0.540	0.515	0.842
0.906	0.358	0.868	0.716	0.570	0.433	0.842	0.872	0.493	0.839	0.996	0.710	0.364	0.796
0.540	0.365	0.406	0.421	0.523	0.685	0.568	0.745	0.513	0.399	0.649	0.912	0.389	0.568
0.429	0.385	0.962	0.716	0.520	0.552	0.996	0.872	0.576	0.554	0.957	0.704	0.980	0.874
0.518	0.426	0.962	0.518	0.948	0.685	0.387	0.996	0.493	0.839	0.540	0.710	0.358	0.387
0.636	0.385	0.366	0.906	0.570	0.744	0.796	0.382	0.513	0.361	0.855	0.521	0.658	0.874
0.448	0.540	0.385	0.361	0.852	0.521	0.685	0.996	0.978	0.789	0.402	0.693	0.553	0.693
0.674	0.556	0.776	1.000	0.986	0.540	0.672	0.669	0.333	0.489	0.513	0.513	0.614	0.513
0.996	0.602	0.358	0.361	0.995	0.704	0.789	0.996	0.359	0.744	0.394	0.440	0.521	0.958
0.712	0.710	0.358	0.693	0.534	0.540	0.433	0.866	0.978	0.789	0.610	0.693	0.540	0.361
0.567	0.602	0.980	0.440	0.852	0.954	0.984	0.361	0.333	0.433	0.431	0.361	0.704	0.958
0.572	0.951	0.433	0.361	0.862	0.361	0.513	0.671	0.336	0.433	0.651	0.665	0.847	0.665
0.449	0.839	0.839	1.000	0.855	0.667	0.839	1.000	0.362	0.620	0.953	0.547	0.367	0.714
0.547	0.693	0.839	0.430	0.589	0.361	0.361	0.869	1.000	1.000	0.541	1.000	0.958	0.430
0.681	0.839	0.388	0.667	1.000	0.508	0.693	0.362	0.336	0.388	0.858	0.430	0.449	0.714
0.676	0.877	0.399	0.361	0.991	0.440	0.569	0.671	0.824	0.588	0.631	0.755	0.392	0.525
0.926	0.718	0.399	0.693	0.538	1.000	0.548	0.747	0.336	0.433	0.762	0.665	0.817	0.548
0.781	0.877	0.789	0.440	0.862	0.554	0.665	0.440	1.000	0.789	0.730	0.548	0.488	0.525
0.714	0.799	1.000	0.430	0.535	0.440	0.388	0.869	0.362	0.620	0.527	0.547	0.361	0.367
0.568	1.000	0.361	0.667	0.855	0.681	0.799	0.362	0.824	0.508	0.824	0.667	0.667	1.000
